# Variable Smoothing for Convex Optimization Problems Using Stochastic Gradients

**DOI:** 10.1007/s10915-020-01332-8

**Published:** 2020-10-22

**Authors:** Radu Ioan Boţ, Axel Böhm

**Affiliations:** Faculty of Mathematics, University of Vienna, Oskar-Morgenstern-Platz 1, 1090 Vienna, Austria

**Keywords:** Structured convex optimization problem, Variable smoothing algorithm, Convergence rate, Stochastic gradients, 90C25, 90C15, 65Y20

## Abstract

We aim to solve a structured convex optimization problem, where a nonsmooth function is composed with a linear operator. When opting for full splitting schemes, usually, primal–dual type methods are employed as they are effective and also well studied. However, under the additional assumption of Lipschitz continuity of the nonsmooth function which is composed with the linear operator we can derive novel algorithms through regularization via the Moreau envelope. Furthermore, we tackle large scale problems by means of stochastic oracle calls, very similar to stochastic gradient techniques. Applications to total variational denoising and deblurring, and matrix factorization are provided.

## Introduction

The problem at hand is the following structured convex optimization problem

1 $$\begin{aligned} \min _{x \in {\mathcal {H}}{}} f(x) + g(Kx), \end{aligned}$$

for real Hilbert spaces ${\mathcal {H}}$ and ${\mathcal {G}}$, $f: {\mathcal {H}}\rightarrow {{\overline{{\mathbb {R}}}}:= {\mathbb {R}}\cup \{\pm \infty \}}$ a proper, convex and lower semicontinuous function, $g:{\mathcal {G}}\rightarrow {\mathbb {R}}$ a, possibly nonsmooth, convex and Lipschitz continuous function, and $K: {\mathcal {H}}\rightarrow {\mathcal {G}}$ a linear continuous operator.

Our aim will be to devise an algorithm for solving (1) following the *full splitting* paradigm (see [5, 6, 8, 9, 15, 17, 29]). In other words, we allow only proximal evaluations for simple nonsmooth functions, but no proximal evaluations for compositions with linear continuous operators, like, for instance, for $g \circ K$.

We will accomplish this feat by the means of a *smoothing strategy*, which, for the purpose of this paper, means, making use of the Moreau-Yosida approximation. The approach can be described as follows: we “smooth” *g*, i.e. we replace it by its Moreau envelope, and solve the resulting optimization problem by an *accelerated proximal-gradient algorithm* (see [3, 13, 21]). This approach is similar to those in [7, 10, 11, 20, 22], where a convergence rate of ${\mathcal {O}}\Big (\frac{\log (k)}{k}\Big )$ is proved. However, our techniques (for the deterministic case) resemble more the ones in [28], where an improved rate of ${\mathcal {O}}(\frac{1}{k})$ is shown, with the most notable difference to our work is that we use a simpler stepsize and treat the stochastic case.

The only other family of methods able to solve problems of type (1) are the so called primal–dual algorithms, first and foremost the *primal–dual hybrid gradient (PDHG)* introduced in [15]. In comparison, this method does not need the Lipschitz continuity of *g* in order to prove convergence. However, in this very general case, convergence rates can only be shown for the so-called *restricted primal–dual gap* function. In order to derive from here convergence rates for the primal objective function, either Lipschitz continuity of *g* or finite dimensionality of the problem plus the condition that *g* must have full domain are necessary (see, for instance, [5, Theorem 9]). This means, that for infinite dimensional problems the assumptions required by both, PDHG and our method, for deriving convergence rates for the primal objective function are in fact equal, but for finite dimensional problems the assumption of PDHG are weaker. In either case, however, we are able to prove these rates for the sequence of iterates ${(x_{k})}_{k \ge 1}$ itself whereas PDHG only has them for the sequence of so-called *ergodic iterates*, i.e. ${(\frac{1}{k}\sum _{i=1}^{k} x_{i})}_{k \ge 1}$, which is naturally undesirable as the averaging slows the convergence down. Furthermore, we do not show any convergence for the iterates as these are notoriously hard to obtain for accelerated method whereas PDHG gets these in the strongly convex setting via standard fixed point arguments (see e.g. [29]).

Furthermore, we will also consider the case where only a stochastic oracle of the proximal operator of *g* is available to us. This setup corresponds e.g. to the case where the objective function is given as

2 $$\begin{aligned} \min _{x \in {\mathcal {H}}} f(x) + \sum _{i=1}^{m} g_{i}(K_{i}x), \end{aligned}$$

where, for $i=1,\dots ,m$, ${\mathcal {G}}_i$ are real Hilbert spaces, $g_i : {\mathcal {G}}_i \rightarrow {\mathbb {R}}$ are convex and Lipschitz continuous functions and $K_i : {\mathcal {H}}\rightarrow {\mathcal {G}}_i$ are linear continuous operators, but the number of summands being large we wish to not compute all proximal operators of all $g_i, i=1, \dots , m$, for purpose of making iterations cheaper to compute.

For the finite sum case (2), there exist algorithms of similar spirit such as those in [14, 24]. Some algorithms do in fact deal with a similar setup of stochastic gradient like evaluations, see [26], but only for smooth terms in the objective function.

In Sect. 2 we will cover the preliminaries about the Moreau-Yosida envelope as well as useful identities and estimates connected to it. In Sect. 3 we will deal with the deterministic case and prove a convergence rate of ${\mathcal {O}}(\frac{1}{k})$ for the function values at the iterates. Next up, in Sect. 4, we will consider the stochastic case as described above and prove a convergence rate of ${\mathcal {O}}\left( \frac{\log (k)}{\sqrt{k}}\right)$. Last but not least, we will look at some numerical examples in image processing in Sect. 5.

It is important to note that the proof for the deterministic setting differs surprisingly from the one for the stochastic setting. The technique for the stochastic setting is less refined in the sense that there is no coupling between the smoothing parameter and the extrapolation parameter. Where as this technique works also works for the deterministic setting it gives a worse convergence rate of ${\mathcal {O}}\left( \frac{\log {k}}{k}\right)$. The tight coupling of the two sequences of parameters, however does not work in the proof of the stochastic algorithm as it does not allow for the particular choice of the smoothing parameters needed there.

## Preliminaries

In the main problem (1), the nonsmooth function regularizer *g* is supposed to be Lipschitz continuous. This assumption is necessary to ensure our main convergence results, however, many of the preliminary lemmata of this section hold true similarly if the function is only assumed to be lower semicontinuous. We will point this out in every statement of this section individually.

### Definition 2.1

For a proper, convex and lower semicontinuous function $g: {\mathcal {H}}\rightarrow {\overline{{\mathbb {R}}}}$, its convex conjugate is denoted by $g^*$ defined as a function from ${\mathcal {H}}$ to ${\overline{{\mathbb {R}}}}$, given by

$$\begin{aligned} g^*(x) : = \sup _{p \in {\mathcal {H}}{}}\left\{ \left\langle x, p \right\rangle - g(p) \right\} \quad \forall x \in {\mathcal {H}}. \end{aligned}$$

As mentioned in the introduction, we want to *smooth* a nonsmooth function by considering its Moreau envelope. The next definition will clarify exactly what object we are talking about.

### Definition 2.2

For a proper, convex and lower semicontinuous function $g: {\mathcal {H}}\rightarrow {\overline{{\mathbb {R}}}}$, its Moreau envelope with the parameter $\mu \ge 0$ is defined as a function from ${\mathcal {H}}$ to ${\mathbb {R}}$, given by

$$\begin{aligned} {}^{\mu _{}}g(\cdot ) := {\left( g^* + \frac{\mu }{2}\Vert \cdot \Vert ^2 \right) }^*(\cdot ) = \sup _{p \in {\mathcal {H}}}\left\{ \left\langle \cdot , p \right\rangle - g^*(p) - \frac{\mu }{2}\Vert p \Vert ^2\right\} . \end{aligned}$$

From this definition, however, it is not completely evident that the Moreau envelope indeed fulfills its purpose in being a smooth representation of the original function. The next lemma will remedy this fact.

### Lemma 2.1

(see [2, Proposition 12.29]) Let $g: {\mathcal {H}}\rightarrow {\overline{{\mathbb {R}}}}$ be a proper, convex and lower semicontinuous function and $\mu > 0$. Then its Moreau envelope is Fréchet differentiable on ${\mathcal {H}}$. In particular, the gradient itself is given by

$$\begin{aligned} \nabla ({}^{\mu _{}}g)(x) = \frac{1}{\mu }\left( x - {\text {prox}}^{}_{\mu g}\left( x \right) \right) = {\text {prox}}^{}_{\frac{1}{\mu }g^*}\left( \frac{x}{\mu } \right) \quad \forall x \in {\mathcal {H}}{} \end{aligned}$$

and is $\mu ^{-1}$-Lipschitz continuous.

In particular, for all $\mu > 0$, a gradient step with respect to the Moreau envelope corresponds to a proximal step

$$\begin{aligned} x - \mu \nabla ({}^{\mu _{}}g)(x) = {\text {prox}}^{}_{\mu g}\left( x \right) \quad \forall x \in {\mathcal {H}}. \end{aligned}$$

The previous lemma establishes two things. Not only does it clarify the smoothness of the Moreau envelope, but it also gives a way of computing its gradient. Obviously, a smooth representation whose gradient we would not be able to compute would not be any good.

As mentioned in the introduction, we want to smooth the nonsmooth summand of the objective function which is composed with the linear operator as this can be considered the crux of problem (1). The function $g \circ K$ will be *smoothed* via considering instead ${}^{\mu _{}}g \circ K : {\mathcal {H}}\rightarrow {\mathbb {R}}$. Clearly, by the chain rule, this function is continuously differentiable with gradient given for every $x \in {\mathcal {H}}$ by

$$\begin{aligned} \nabla \left( {}^{\mu _{}}g \circ K \right) (x) = K^* \nabla \left( {}^{\mu _{}}g \right) (K x) = \frac{1}{\mu }K^*\left( Kx - {\text {prox}}^{}_{\mu g}\left( Kx \right) \right) = K^* {\text {prox}}^{}_{\frac{1}{\mu }g^*}\left( \frac{Kx}{\mu } \right) , \end{aligned}$$

and is thus Lipschitz continuous with Lipschitz constant $\frac{\Vert K \Vert ^2}{\mu }$, where $\Vert K \Vert$ denotes the operator norm of *K*.

Lipschitz continuity will play an integral role in our investigations, as can be seen by the following lemmas.

### Lemma 2.2

(see [4, Proposition 4.4.6]) Let $g:{\mathcal {H}}\rightarrow {\mathbb {R}}$ be a convex and $L_g$-Lipschitz continuous function. Then, the domain of its Fenchel conjugate is bounded, i.e.

$$\begin{aligned} dom \, g^* \subseteq B(0,L_g), \end{aligned}$$

where $B(0,L_g)$ denotes the open ball with radius $L_g$ around the origin.

The Moreau envelope even preserves the Lipschitzness of the original function.

### Lemma 2.3

(see [18, Lemma 2.1]) Let $g: {\mathcal {H}}\rightarrow {\mathbb {R}}$ be a convex and $L_g$-Lipschitz continuous function. Then its Moreau envelope ${}^{\mu _{}}g$ is $L_g$-Lipschitz as well, i.e.

$$\begin{aligned} |{}^{\mu _{}}g(x) - {}^{\mu _{}}g(y) |\le L_{g}\Vert x-y \Vert \quad \forall x,y \in {\mathcal {H}}. \end{aligned}$$

### Proof

We observe that for all $x \in {\mathcal {H}}$

$$\begin{aligned} \nabla {}^{\mu _{}}g(x) \in \partial g({\text {prox}}^{}_{\mu g}\left( x \right) ). \end{aligned}$$

Therefore we can bound the gradient norm

3 $$\begin{aligned} \Vert \nabla {}^{\mu _{}}g(x) \Vert \le \sup \{ \Vert v \Vert : y \in {\mathcal {H}}, v \in \partial g(y) \} \le L_g \quad \forall x \in {{{\mathcal {H}}}}, \end{aligned}$$

where we used in the last step that the Lipschitz continuity of *g*. The statement follows from the mean-value theorem. $\square$

The following lemmata are not new, but we provide proofs anyways in order to remain self-contained and to shed insight on how to use the Moreau envelope for the interested reader.

### Lemma 2.4

(see [28, Lemma 10 (a)]) Let $g: {\mathcal {H}}\rightarrow {\overline{{\mathbb {R}}}}$ be proper, convex and lower semicontinuous. The maximizing argument in the definition of the Moreau-Yosida envelope is given by its gradient, i.e. for $\mu >0$ it holds that

$$\begin{aligned} \mathop {\mathrm {arg\, max}}\limits _{p \in {\mathcal {H}}}\left\{ \left\langle \cdot , p \right\rangle - g^*(p) - \frac{\mu }{2}\Vert p \Vert ^2\right\} = \nabla {}^{\mu _{}}g(\cdot ). \end{aligned}$$

### Proof

Let $x \in {\mathcal {H}}$ be fixed. It holds

$$\begin{aligned} \begin{aligned} \mathop {\mathrm {arg\, max}}\limits _{p \in {\mathcal {H}}}\left\{ \langle x, p \rangle - g^*(p) - \frac{\mu }{2}\Vert p \Vert ^2\right\} =&\mathop {\mathrm {arg\, max}}\limits _{p \in {\mathcal {H}}}\left\{ -\frac{1}{2\mu }\Vert x \Vert ^2 + \langle x, p \rangle - \frac{\mu }{2}\Vert p \Vert ^2 - g^*(p) \right\} \\ =&\mathop {\mathrm {arg\, max}}\limits _{p \in {\mathcal {H}}}\left\{ -\frac{\mu }{2}\left\Vert \frac{x}{\mu } - p \right\Vert ^2 - g^*(p) \right\} \\ =&\mathop {\mathrm {arg\, min}}\limits _{p \in {\mathcal {H}}}\left\{ g^*(p) + \frac{\mu }{2}\left\Vert \frac{x}{\mu } - p \right\Vert ^2 \right\} \\ =&{\text {prox}}^{}_{\frac{1}{\mu }g^*}\left( \frac{x}{\mu } \right) \end{aligned} \end{aligned}$$

and the conclusion follows by using Lemma 2.1. $\square$

### Lemma 2.5

(see [28, Lemma 10 (a)]) For a proper, convex and lower semicontinuous function $g: {\mathcal {H}}\rightarrow {\overline{{\mathbb {R}}}}$ and every $x \in {\mathcal {H}}$ we can consider the mapping from $(0, +\infty )$ to ${\mathbb {R}}$ given by

4 $$\begin{aligned} \mu \mapsto {}^{\mu _{}} g(x). \end{aligned}$$

This mapping is convex and differentiable and its derivative is given by

$$\begin{aligned} \frac{\partial }{\partial \mu } {}^{\mu _{}} g(x) = - \frac{1}{2}\Vert \nabla {}^{\mu _{}} g(x) \Vert ^2 \qquad \forall x \in {\mathcal {H}}\ \forall \mu \in (0, +\infty ). \end{aligned}$$

### Proof

Let $x \in {\mathcal {H}}$ be fixed. From the definition of the Moreau-Yosida envelope we can see that the mapping given in (4) is a pointwise supremum of functions which are linear in $\mu$. It is therefore convex. Furthermore, since the objective function is strongly concave, this supremum is uniquely attained at $\nabla {}^{\mu _{}} g(x) = \mathop {\mathrm {arg\, max}}\limits _{p \in {\mathcal {H}}} \left\{ \left\langle x, p \right\rangle - g^*(p) - \frac{\mu }{2}\Vert p \Vert ^2\right\}$. According to the Danskin Theorem, the function $\mu \mapsto {}^{\mu _{}} g(x)$ is differentiable and its gradient is given by

$$\begin{aligned} \begin{aligned} \frac{\partial }{\partial \mu } {}^{\mu _{}} g(x) =&\frac{\partial }{\partial \mu } \sup _{p \in {\mathcal {H}}}\left\{ \langle x, p \rangle - g^*(p) - \frac{\mu }{2}\Vert p \Vert ^2\right\} \\ =&-\frac{1}{2}\Vert \nabla {}^{\mu _{}} g(x) \Vert ^2 \quad \forall \mu \in (0, +\infty ). \end{aligned} \end{aligned}$$

$$\square $$

### Lemma 2.6

( [28, Lemma 10 (b)]) Let $g: {\mathcal {H}}\rightarrow {\overline{{\mathbb {R}}}}$ be proper, convex and lower semicontinuous. For $\mu _{1}, \mu _{2} > 0$ and every $x \in {\mathcal {H}}$ it holds

5 $$\begin{aligned} {}^{\mu _{1}} g (x) \le {}^{\mu _{2}}g(x) + (\mu _2 - \mu _1)\frac{1}{2} \Vert \nabla {}^{\mu _{1}} g(x) \Vert ^2. \end{aligned}$$

If *g* is additionally $L_{g}$-Lipschitz and if $\mu _{2}\ge \mu _{1} > 0$, then

6 $$\begin{aligned} {}^{\mu _{2}}g(x) \le {}^{\mu _{1}}g(x) \le {}^{\mu _{2}}g(x) + (\mu _2 - \mu _1)\frac{L_g^2}{2}. \end{aligned}$$

### Proof

Let $x \in {\mathcal {H}}$ be fixed. Via Lemma 2.5 we know that the map $\mu \mapsto {}^{\mu _{}} g (x)$ is convex and differentiable. We can therefore use the gradient inequality to deduce that

$$\begin{aligned} \begin{aligned} {}^{\mu _{2}}g(x) \ge&\, {}^{\mu _{1}}g(x) + (\mu _{2} - \mu _{1}) \left( \frac{\partial }{\partial \mu } {}^{\mu _{}}g(x) \Bigr |_{\mu = \mu _{1}} \right) \\ =&\, {}^{\mu _{1}}g(x) - (\mu _{2} - \mu _{1})\frac{1}{2} \Vert \nabla {}^{\mu _{1}}g(x) \Vert ^2, \end{aligned} \end{aligned}$$

which is exactly the first statement of the lemma. The first inequality of (6) follows directly from the definition of the Moreau envelope and the second one from (5) and (3). $\square$

By applying a limiting argument it is easy to see that (6) implies that for any $\mu >0$

7 $$\begin{aligned} {}^{\mu _{}}g(x) \le g(x) \le {}^{\mu _{}}g(x) + \mu \frac{L_g^2}{2}, \end{aligned}$$

which shows that the Moreau envelope is always a lower approximation the original function.

### Lemma 2.7

(see [28, Lemma 10 (c)]) Let $g: {\mathcal {H}}\rightarrow {\overline{{\mathbb {R}}}}$ be proper, convex and lower semicontinuous. Then, for $\mu > 0$ and every $x,y \in {\mathcal {H}}$ we have that

$$\begin{aligned} {}^{\mu _{}} g(x) + \left\langle \nabla {}^{\mu _{}}g(x), y-x \right\rangle \le g(y) - \frac{\mu }{2} \Vert \nabla {}^{\mu _{}}g(x) \Vert ^2. \end{aligned}$$

### Proof

Using Lemma 2.4 and the definition of the Moreau-Yosida envelope we get that

$$\begin{aligned} \begin{aligned} {}^{\mu _{}}g(x) + \left\langle \nabla {}^{\mu _{}}g(x), y - x \right\rangle =&\left\langle x, \nabla {}^{\mu _{}}g(x) \right\rangle - g^*(\nabla {}^{\mu _{}}g(x)) \\&- \frac{\mu }{2}\Vert \nabla {}^{\mu _{}}g(x) \Vert ^2 + \left\langle \nabla {}^{\mu _{}}g(x), y-x \right\rangle \\ =&\left\langle \nabla {}^{\mu _{}}g(x), y \right\rangle - g^*(\nabla {}^{\mu _{}}g(x)) - \frac{\mu }{2}\Vert \nabla {}^{\mu _{}}g(x) \Vert ^2 \\ \le&\ \sup _{p \in {\mathcal {H}}}\{\left\langle p, y \right\rangle - g^*(p) \} - \frac{\mu }{2}\Vert \nabla {}^{\mu _{}}g(x) \Vert ^2 \\ =&\ g(y) - \frac{\mu }{2}\Vert \nabla {}^{\mu _{}}g(x) \Vert ^2. \end{aligned} \end{aligned}$$

$$\square $$

In the convergence proof of Lemma 3.3 we will need the inequality in the above lemma at the points *Kx* and *Ky*, namely

8 $$\begin{aligned} \begin{aligned} g(Ky) - \frac{\mu }{2} \Vert \nabla {}^{\mu _{}}g(Kx) \Vert ^2&\ge {}^{\mu _{}} g(Kx) + \left\langle \nabla {}^{\mu _{}}g(Kx), Ky-Kx \right\rangle \\&= {}^{\mu _{}} g(Kx) + \left\langle K^{*}\nabla {}^{\mu _{}}g(Kx), y-x \right\rangle \\&= {}^{\mu _{}} g(Kx) + \left\langle \nabla ({}^{\mu _{}}g \circ K)(x), y-x \right\rangle \quad \forall x,y \in {\mathcal {H}}. \end{aligned} \end{aligned}$$

The following lemma is a standard result for convex and Fréchet differentiable functions.

### Lemma 2.8

(see [23]) For a convex and Fréchet differentiable function $h: {\mathcal {H}}\rightarrow {\mathbb {R}}$ with $L_{h}$-Lipschitz continuous gradient we have that

$$\begin{aligned} h(x) + \left\langle \nabla h(x), y-x \right\rangle \le h(y) - \frac{1}{2L_{h}} \Vert \nabla h(x) - \nabla h(y) \Vert ^2 \quad \forall x,y \in {\mathcal {H}}. \end{aligned}$$

By applying Lemma 2.8 with ${}^{\mu _{}}g$, *Kx* and *Ky* instead of *h*, *x* and *y* respectively, we obtain

9 $$\begin{aligned} {}^{\mu _{}}g(Kx) + \left\langle \nabla ({}^{\mu _{}}g \circ K)(x), y-x \right\rangle \le {}^{\mu _{}}g(Ky) - \frac{\mu }{2} \Vert \nabla {}^{\mu _{}}g(Kx) - \nabla {}^{\mu _{}}g(Ky) \Vert ^2 \quad \forall x,y \in {\mathcal {H}}. \end{aligned}$$

The following technical result will be used in the proof of the convergence statement.

### Lemma 2.9

For $\alpha \in (0,1)$ and every $x,y \in {\mathcal {H}}$ we have that

$$\begin{aligned} (1 - \alpha )\Vert x - y \Vert ^2 + \alpha \Vert y \Vert ^2 \ge \alpha (1-\alpha )\Vert x \Vert ^2. \end{aligned}$$

## Deterministic Method

### Problem 3.1

The problem at hand reads

$$\begin{aligned} \min _{x \in {\mathcal {H}}{}}\, F(x): =f(x) + g(Kx), \end{aligned}$$

for a proper, convex and lower semicontinuous function $f: {\mathcal {H}}\rightarrow {\overline{{\mathbb {R}}}}$, a convex and $L_g$-Lipschitz continuous $(L_g >0)$ function $g:{\mathcal {G}}\rightarrow {\mathbb {R}}$, and a nonzero linear continuous operator $K : {\mathcal {H}}\rightarrow {\mathcal {G}}$.

The idea of the algorithm which we propose to solve (1) is to smooth *g* and then to solve the resulting problem by means of an accelerated proximal-gradient method.

### Algorithm 3.1

(Variable Accelerated SmooThing (VAST)) Let $y_0 = x_0 \in {\mathcal {H}}, {(\mu _{k})}_{k \ge 0} \! \subseteq (0,+\infty )$, and ${(t_{k})}_{k \ge 1}$ a sequence of real numbers with $t_1=1$ and $t_{k} \ge 1$ for every $k\ge 2$. Consider the following iterative scheme

$$\begin{aligned} (\forall k \ge 1) \quad \left\lfloor \begin{array}{l} L_{k} = \frac{\Vert K \Vert ^2}{\mu _{k}} \\ \gamma _{k} = \frac{1}{L_{k}} \\ x_{k} = {\text {prox}}^{}_{\gamma _{k}f}\left( y_{k-1} - \gamma _{k} K^*{\text {prox}}^{}_{\frac{1}{\mu _{k}}g^*}\left( \frac{K y_{k-1}}{\mu _{k}} \right) \right) \\ y_{k} = x_{k} + \frac{t_{k}-1}{t_{k+1}}(x_{k} - x_{k-1}). \end{array}\right. \end{aligned}$$

### Remark 3.1

The assumption $t_{1} = 1$ can be removed but guarantees easier computation and is also in line with classical choices of ${(t_{k})}_{k \ge 1}$ in [13, 21].

### Remark 3.2

The sequence ${(u_{k})}_{k \ge 1}$ given by

$$\begin{aligned} u_{k} := x_{k-1} + t_{k}(x_{k} - x_{k-1}) \quad \forall k \ge 1, \end{aligned}$$

despite not appearing in the algorithm, will feature a prominent role in the convergence proof. Due to the convention $t_1 = 1$ we have that

$$\begin{aligned} u_{1} := x_{0} + t_{1}(x_{1} - x_{0}) = x_{1}. \end{aligned}$$

We also denote

$$\begin{aligned} F^{k} = f + {}^{\mu _{k}}g \circ K \quad \forall k \ge 0. \end{aligned}$$

The next theorem is the main result of this section and it will play a fundamental role when proving a convergence rate of ${\mathcal {O}}(\frac{1}{k})$ for the sequence ${(F(x_k))}_{k \ge 0}$.

### Theorem 3.1

Consider the setup of Problem 3.1 and let ${(x_{k})}_{k \ge 0}$ and ${(y_{k})}_{k \ge 0}$ be the sequences generated by Algorithm 3.1. Assume that for every $k\ge 1$

$$\begin{aligned} \mu _{k} - \mu _{k+1} - \frac{\mu _{k+1}}{t_{k+1}} \le 0 \end{aligned}$$

and

$$\begin{aligned} \left( 1 - \frac{1}{t_{k+1}} \right) \gamma _{k+1} t_{k+1}^2 = \gamma _{k}t_{k}^2. \end{aligned}$$

Then, for every optimal solution $x^*$ of Problem 3.1, it holds

$$\begin{aligned} F(x_{N}) - F(x^*) \le \frac{\Vert x_{0} - x^*\Vert ^2 }{2\gamma _{N} t_{N}^{2}} + \mu _{N}\frac{L_{g}^2}{2} \quad \forall N \ge 1. \end{aligned}$$

The proof of this result relies on several partial results which we will prove as follows.

### Lemma 3.1

The following statement holds for every $z \in {\mathcal {H}}$ and every $k\ge 0$

$$\begin{aligned}&F^{k+1}(x_{k+1}) + \frac{1}{2 \gamma _{k+1}} \Vert x_{k+1} - z \Vert ^2 \\&\quad \le f(z) + {}^{\mu _{k+1}}g(Ky_{k}) + \left\langle \nabla ({}^{\mu _{k+1}} g \circ K ) (y_{k}), z - y_{k}\right\rangle + \frac{1}{2 \gamma _{k+1}} \Vert z - y_{k} \Vert ^2. \end{aligned}$$

### Proof

Let $k\ge 0$ be fixed. Since, by the definition of the proximal map, $x_{k+1}$ is the minimizer of a $\frac{1}{\gamma _{k+1}}$-strongly convex function we know that for every $z \in {\mathcal {H}}$

$$\begin{aligned}&f(x_{k+1}) + {}^{\mu _{k+1}}g(Ky_{k}) + \left\langle \nabla ({}^{\mu _{k+1}} g \circ K ) (y_{k}), x_{k+1} - y_{k}\right\rangle + \frac{1}{2 \gamma _{k+1}} \Vert x_{k+1} - y_{k} \Vert ^2 + \\&\quad \frac{1}{2 \gamma _{k+1}} \Vert x_{k+1} - z \Vert ^2 \\&\quad \le f(z) + {}^{\mu _{k+1}}g(Ky_{k}) + \left\langle \nabla ({}^{\mu _{k+1}} g \circ K ) (y_{k}), z - y_{k}\right\rangle + \frac{1}{2 \gamma _{k+1}} \Vert z - y_{k} \Vert ^2. \end{aligned}$$

Next we use the $L_{k+1}$-smoothness of ${}^{\mu _{k+1}} g \circ K$ and the fact that $\frac{1}{\gamma _{k+1}} = L_{k+1}$ to deduce

$$\begin{aligned}&f(x_{k+1}) + {}^{\mu _{k+1}}g(Kx_{k+1}) + \frac{1}{2 \gamma _{k+1}} \Vert x_{k+1} - z \Vert ^2 \\&\quad \le f(z) + {}^{\mu _{k+1}}g(Ky_{k}) + \left\langle \nabla ({}^{\mu _{k+1}} g \circ K ) (y_{k}), z - y_{k}\right\rangle + \frac{1}{2 \gamma _{k+1}} \Vert z - y_{k} \Vert ^2.&\end{aligned}$$

$$\square $$

### Lemma 3.2

Let $x^*$ be an optimal solution of Problem 3.1. Then it holds

$$\begin{aligned} \gamma _1 (F^{1}(x_{1})- F(x^*)) + \frac{1}{2}\Vert u_{1} - x^* \Vert ^2 \le \frac{1}{2} \Vert x^* - x_{0} \Vert ^2. \end{aligned}$$

### Proof

We use the gradient inequality to deduce that for every $z \in {\mathcal {H}}$ and every $k \ge 0$

$$\begin{aligned} {}^{\mu _{k+1}}g(Ky_{k}) + \left\langle \nabla ({}^{\mu _{k+1}} g \circ K ) (y_{k}), z - y_{k}\right\rangle \le {}^{\mu _{k+1}}g(Kz) \le g(Kz) \end{aligned}$$

and plug this into the statement of Lemma 3.1 to conclude that

$$\begin{aligned} F^{k+1}(x_{k+1}) + \frac{1}{2 \gamma _{k+1}} \Vert x_{k+1} - z \Vert ^2 \le F(z) + \frac{1}{2 \gamma _{k+1}} \Vert z - y_{k} \Vert ^2. \end{aligned}$$

For $k=0$ we get that

$$\begin{aligned} F^{1}(x_{1}) + \frac{1}{2 \gamma _{1}} \Vert x_{1} - x^* \Vert ^2 \le F(x^*) + \frac{1}{2 \gamma _{1}} \Vert x^* - y_{0} \Vert ^2. \end{aligned}$$

Now we us the fact that $u_1 = x_1$ and $y_0= x_0$ to obtain the conclusion. $\square$

### Lemma 3.3

Let $x^*$ be an optimal solution of Problem 3.1. The following descent-type inequality holds for every $k \ge 0$

$$\begin{aligned} \begin{aligned}&F^{k+1}(x_{k+1}) - F(x^*) + \frac{\Vert u_{k+1} - x^* \Vert ^2}{2 \gamma _{k+1} t_{k+1}^2} \le \left( 1- \frac{1}{t_{k+1}}\right) \left( F^{k}(x_{k}) - F(x^*)\right) + \frac{\Vert u_{k} - x^* \Vert ^2}{2 \gamma _{k+1} t_{k+1}^2} \\&\quad + \left( 1- \frac{1}{t_{k+1}}\right) \left( \mu _{k} - \mu _{k+1}- \frac{\mu _{k+1}}{t_{k+1}}\right) \Vert \nabla {}^{\mu _{k+1}}g(Kx_{k}) \Vert ^2. \end{aligned} \end{aligned}$$

### Proof

Let $k \ge 0$ be fixed. We apply Lemma 3.1 with $z := \left( 1- \frac{1}{t_{k+1}} \right) x_{k} + \frac{1}{t_{k+1}} x^*$ to deduce that

$$\begin{aligned} \begin{aligned}&F^{k+1}(x_{k+1}) + \frac{\Vert u_{k+1} - x^* \Vert ^2}{2 \gamma _{k+1} t_{k+1}^2} \le f\left( \left( 1- \frac{1}{t_{k+1}}\right) x_{k} + \frac{1}{t_{k+1}} x^* \right) + {}^{\mu _{k+1}}g(Ky_{k}) \\&\quad + \left( 1- \frac{1}{t_{k+1}}\right) \left\langle \nabla ({}^{\mu _{k+1}} g \circ K ) (y_{k}), x_{k} - y_{k}\right\rangle \\&\quad + \frac{1}{t_{k+1}} \left\langle \nabla ({}^{\mu _{k+1}} g \circ K ) (y_{k}), x^* - y_{k}\right\rangle + \frac{1}{2 \gamma _{k+1} t_{k+1}^2} \Vert u_{k} - x^* \Vert ^2. \end{aligned} \end{aligned}$$

Using the convexity of *f* gives

10 $$\begin{aligned} \begin{aligned}&F^{k+1}(x_{k+1}) + \frac{\Vert u_{k+1} - x^* \Vert ^2}{2 \gamma _{k+1} t_{k+1}^2} \le \left( 1- \frac{1}{t_{k+1}}\right) f(x_{k}) + \frac{1}{t_{k+1}}f(x^*) \\&\quad + \left( 1- \frac{1}{t_{k+1}}\right) {}^{\mu _{k+1}}g(Ky_{k}) + \left( 1- \frac{1}{t_{k+1}}\right) \left\langle \nabla ({}^{\mu _{k+1}} g \circ K ) (y_{k}), x_{k} - y_{k}\right\rangle \\&\quad + \frac{1}{t_{k+1}} {}^{\mu _{k+1}}g(Ky_{k}) + \frac{1}{t_{k+1}} \left\langle \nabla ({}^{\mu _{k+1}} g \circ K ) (y_{k}), x^* - y_{k}\right\rangle + \frac{\Vert u_{k} - x^* \Vert ^2}{2 \gamma _{k+1} t_{k+1}^2}. \end{aligned} \end{aligned}$$

Now, we use (8) to deduce that

11 $$\begin{aligned} \begin{aligned}&\frac{1}{t_{k+1}} {}^{\mu _{k+1}}g(Ky_{k}) + \frac{1}{t_{k+1}} \left\langle \nabla ({}^{\mu _{k+1}} g \circ K ) (y_{k}), x^* - y_{k}\right\rangle \\&\quad \le \frac{1}{t_{k+1}} g(Kx^*) - \frac{1}{t_{k+1}}\frac{\mu _{k+1}}{2} \Vert \nabla {}^{\mu _{k+1}}g (Ky_{k}) \Vert ^2 \end{aligned} \end{aligned}$$

and (9) to conclude that

12 $$\begin{aligned} \begin{aligned}&\left( 1- \frac{1}{t_{k+1}}\right) {}^{\mu _{k+1}}g(Ky_{k}) + \left( 1- \frac{1}{t_{k+1}}\right) \left\langle \nabla ({}^{\mu _{k+1}} g \circ K ) (y_{k}), x_{k} - y_{k}\right\rangle \\&\quad \le \left( 1- \frac{1}{t_{k+1}}\right) {}^{\mu _{k+1}}g(Kx_{k}) - \left( 1- \frac{1}{t_{k+1}}\right) \frac{\mu _{k+1}}{2}\Vert \nabla {}^{\mu _{k+1}}g (Ky_{k}) - \nabla {}^{\mu _{k+1}} g (Kx_{k}) \Vert ^2. \end{aligned} \end{aligned}$$

Combining (10), (11) and (12) gives

$$\begin{aligned} \begin{aligned}&F^{k+1}(x_{k+1}) + \frac{\Vert u_{k+1} - x^* \Vert ^2}{2 \gamma _{k+1} t_{k+1}^2} \le \left( 1- \frac{1}{t_{k+1}}\right) {}^{\mu _{k+1}}g(Kx_{k}) + \left( 1- \frac{1}{t_{k+1}}\right) f(x_{k}) \\&\quad + \frac{1}{t_{k+1}} g(Kx^*) + \frac{1}{t_{k+1}}f(x^*) - \left( 1- \frac{1}{t_{k+1}}\right) \frac{\mu _{k+1}}{2}\Vert \nabla {}^{\mu _{k+1}}g(Ky_{k}) - \nabla {}^{\mu _{k+1}} g(Kx_{k}) \Vert ^2\\&\quad - \frac{1}{t_{k+1}}\frac{\mu _{k+1}}{2} \Vert \nabla {}^{\mu _{k+1}}g(Ky_{k}) \Vert ^2 + \frac{\Vert u_{k} - x^* \Vert ^2}{2 \gamma _{k+1} t_{k+1}^2}. \end{aligned} \end{aligned}$$

The first term on the right hand side is ${}^{\mu _{k+1}}g(Kx_{k})$ but we would like it to be ${}^{\mu _{k}}g(Kx_{k})$. Therefore we use Lemma 2.6 to deduce that

13 $$\begin{aligned} \begin{aligned}&F^{k+1}(x_{k+1}) + \frac{\Vert u_{k+1} - x^* \Vert ^2}{2 \gamma _{k+1} t_{k+1}^2} \le \left( 1- \frac{1}{t_{k+1}}\right) {}^{\mu _{k}}g(Kx_{k}) + \left( 1- \frac{1}{t_{k+1}}\right) f(x_{k}) \\&\quad + \frac{1}{t_{k+1}} g(Kx^*) + \frac{1}{t_{k+1}}f(x^*) + \left( 1- \frac{1}{t_{k+1}}\right) (\mu _{k} - \mu _{k+1})\frac{1}{2}\Vert \nabla {}^{\mu _{k+1}}g(Kx_{k}) \Vert ^2 \\&\quad - \left( 1- \frac{1}{t_{k+1}}\right) \frac{\mu _{k+1}}{2}\Vert \nabla {}^{\mu _{k+1}}g(Ky_{k}) - \nabla {}^{\mu _{k+1}}g(Kx_{k}) \Vert ^2 \\&\quad - \frac{1}{t_{k+1}}\frac{\mu _{k+1}}{2} \Vert \nabla {}^{\mu _{k+1}}g(Ky_{k}) \Vert ^2 + \frac{\Vert u_{k} - x^* \Vert ^2}{2 \gamma _{k+1} t_{k+1}^2}. \end{aligned} \end{aligned}$$

Next up we want to estimate all the norms of gradients by using Lemma 2.9 which says that

14 $$\begin{aligned} \begin{aligned}&\left( 1 - \frac{1}{t_{k+1}} \right) \Vert \nabla {}^{\mu _{k+1}}g(Ky_{k}) - \nabla {}^{\mu _{k+1}}g(Kx_{k})\Vert ^2 + \frac{1}{t_{k+1}} \Vert \nabla {}^{\mu _{k+1}}g(Ky_{k}) \Vert ^2 \\&\quad \ge \left( 1 - \frac{1}{t_{k+1}} \right) \frac{1}{t_{k+1}} \Vert \nabla {}^{\mu _{k+1}}g(Kx_{k}) \Vert ^2. \end{aligned} \end{aligned}$$

Combining (13) and (14) gives

$$\begin{aligned} \begin{aligned}&F^{k+1}(x_{k+1}) + \frac{\Vert u_{k+1} - x^* \Vert ^2}{2 \gamma _{k+1} t_{k+1}^2} \le \left( 1- \frac{1}{t_{k+1}}\right) {}^{\mu _{k}}g(Kx_{k}) + \left( 1- \frac{1}{t_{k+1}}\right) f(x_{k}) \\&\quad + \frac{1}{t_{k+1}} g(Kx^*) + \frac{1}{t_{k+1}}f(x^*) + \left( 1- \frac{1}{t_{k+1}}\right) (\mu _{k} - \mu _{k+1})\frac{1}{2}\Vert \nabla {}^{\mu _{k+1}}g(Kx_{k}) \Vert ^2 \\&\quad - \frac{\mu _{k+1}}{2}\left( 1 - \frac{1}{t_{k+1}} \right) \frac{1}{t_{k+1}} \Vert \nabla {}^{\mu _{k+1}}g(Kx_{k}) \Vert ^2 + \frac{\Vert u_{k} - x^* \Vert ^2}{2 \gamma _{k+1} t_{k+1}^2}. \end{aligned} \end{aligned}$$

Now we combine the two terms containing $\Vert \nabla {}^{\mu _{k+1}}g(Kx_{k}) \Vert ^2$ and get that

$$\begin{aligned} \begin{aligned}&F^{k+1}(x_{k+1}) + \frac{\Vert u_{k+1} - x^* \Vert ^2}{2 \gamma _{k+1} t_{k+1}^2} \le \left( 1- \frac{1}{t_{k+1}}\right) {}^{\mu _{k}}g(Kx_{k}) + \left( 1- \frac{1}{t_{k+1}}\right) f(x_{k}) \\&\quad + \frac{1}{t_{k+1}} g(Kx^*) + \frac{1}{t_{k+1}}f(x^*) + \frac{\Vert u_{k} - x^* \Vert ^2}{2 \gamma _{k+1} t_{k+1}^2} \\&\quad + \left( 1- \frac{1}{t_{k+1}}\right) \left( \mu _{k} - \mu _{k+1}- \frac{\mu _{k+1}}{t_{k+1}}\right) \frac{1}{2} \Vert \nabla {}^{\mu _{k+1}}g(Kx_{k}) \Vert ^2. \end{aligned} \end{aligned}$$

By subtracting $F(x^*) = f(x^*) + g(Kx^*)$ on both sides we finally obtain

$$\begin{aligned} \begin{aligned}&F^{k+1}(x_{k+1}) - F(x^*) + \frac{\Vert u_{k+1} - x^* \Vert ^2}{2 \gamma _{k+1} t_{k+1}^2} \le \left( 1- \frac{1}{t_{k+1}}\right) \left( F^{k}(x_{k}) - F(x^*)\right) + \frac{\Vert u_{k} - x^* \Vert ^2}{2 \gamma _{k+1} t_{k+1}^2} \\&\quad + \left( 1- \frac{1}{t_{k+1}}\right) \left( \mu _{k} - \mu _{k+1}- \frac{\mu _{k+1}}{t_{k+1}}\right) \frac{1}{2} \Vert \nabla {}^{\mu _{k+1}}g(Kx_{k}) \Vert ^2. \end{aligned} \end{aligned}$$

$$\square $$

Now we are in the position to prove Theorem 3.1.

### Proof of Theorem 3.1

We start with the statement of Lemma 3.3 and use the assumption that

$$\begin{aligned} \mu _{k} - \mu _{k+1} - \frac{\mu _{k+1}}{t_{k+1}} \le 0 \end{aligned}$$

to make the last term in the inequality disappear for every $k \ge 0$

$$\begin{aligned} \begin{aligned}&F^{k+1}(x_{k+1}) - F(x^*) + \frac{\Vert u_{k+1} - x^* \Vert ^2}{2 \gamma _{k+1} t_{k+1}^2} \!\\&\quad \le \left( 1- \frac{1}{t_{k+1}}\right) \left( F^{k}(x_{k}) - F(x^*)\right) + \frac{\Vert u_{k} - x^* \Vert ^2}{2 \gamma _{k+1} t_{k+1}^2}. \end{aligned} \end{aligned}$$

Now we use the assumption that

$$\begin{aligned} \left( 1 - \frac{1}{t_{k+1}} \right) \gamma _{k+1} t_{k+1}^2 = \gamma _{k}t_{k}^2 \end{aligned}$$

to get that for every $k \ge 0$

15 $$\begin{aligned}&\gamma _{k+1}t_{k+1}^2(F^{k+1}(x_{k+1}) - F(x^*)) + \frac{\Vert u_{k+1} - x^* \Vert ^2}{2}\nonumber \\&\quad \le \gamma _{k}t_{k}^2 (F^{k}(x_{k}) - F(x^*)) + \frac{\Vert u_{k} - x^* \Vert ^2}{2}. \end{aligned}$$

Let $N \ge 2$. Summing (15) from $k=1$ to $N-1$ and getting rid of the nonnegative term $\Vert u_{N} - x^* \Vert ^2$ gives

$$\begin{aligned} \gamma _{N} t_{N}^{2}(F^{N}(x_{N}) - F(x^*) ) \le \gamma _{1}(F^{1}(x_{1}) - F(x^*)) + \frac{\Vert u_{1}- x^* \Vert ^2}{2} \quad \forall N \ge 2. \end{aligned}$$

Since $t_1=1$, the above inequality is fulfilled also for $N=1$. Using Lemma 3.2 shows that

$$\begin{aligned} F^{N}(x_{N}) - F(x^*) \le \frac{\Vert x_{0} - x^*\Vert ^2 }{\gamma _{N} t_{N}^{2}} \quad \forall N \ge 1. \end{aligned}$$

The above inequality, however, is still in terms of the smoothed objective function. In order to go to the actual objective function we apply (7) and deduce that

$$\begin{aligned} F(x_{N}) - F(x^*) \le F^{N}(x_{N}) - F(x^*) + \mu _{N}\frac{L_{g}^2}{2} \le \frac{\Vert x_{0} - x^*\Vert ^2}{2\gamma _{N} t_{N}^{2}} + \mu _{N}\frac{L_{g}^2}{2} \quad \forall N \ge 1. \end{aligned}$$

$$\square $$

### Corollary 3.1

By choosing the parameters ${(\mu _{k})}_{k\ge 1}, {(t_{k})}_{k\ge 1}, {(\gamma _{k})}_{k\ge 1}$ in the following way,

$$\begin{aligned} t_1=1, \quad \mu _1 = b\Vert K \Vert ^2, \ \text {for} \ b >0, \end{aligned}$$

and for every $k \ge 1$

16 $$\begin{aligned} t_{k+1} := \sqrt{t_{k}^2 + 2 t_{k}}, \quad \mu _{k+1} := \mu _k \frac{t_{k}^2}{t_{k+1}^2 - t_{k+1}}, \quad \gamma _k := \frac{\mu _k}{\Vert K\Vert ^2}, \end{aligned}$$

they fulfill

17 $$\begin{aligned} \mu _{k} - \mu _{k+1} - \frac{\mu _{k+1}}{t_{k+1}} \le 0 \end{aligned}$$

and

18 $$\begin{aligned} \left( 1 - \frac{1}{t_{k+1}} \right) \gamma _{k+1} t_{k+1}^2 = \gamma _{k}t_{k}^2 \end{aligned}$$

For this choice of the parameters we have that

$$\begin{aligned} F(x_{N}) - F(x^*) \le \frac{\Vert x_{0} - x^*\Vert ^2 }{b (N+1)} + \frac{bL_{g}^2\Vert K \Vert ^2}{(N+1)} \exp \left( \frac{4 \pi ^2}{6}\right) \quad \forall N\ge 1. \end{aligned}$$

### Proof

Since $\gamma _{k}$ and $\mu _{k}$ are a scalar multiple of each other (18) is equivalent to

$$\begin{aligned} \left( 1 - \frac{1}{t_{k+1}} \right) \mu _{k+1} t_{k+1}^2 = \mu _{k} t_{k}^2 \quad \forall k \ge 1 \end{aligned}$$

and further to (by taking into account that $t_{k+1} > 1$ for every $k \ge 1$)

19 $$\begin{aligned} \mu _{k+1} = \mu _{k} \frac{t_{k}^2}{t_{k+1}^2}\frac{t_{k+1}}{t_{k+1} - 1} = \mu _{k} \frac{t_{k}^2}{t_{k+1}^2 - t_{k+1}} \quad \forall k \ge 1. \end{aligned}$$

Our update choice in (16) for the sequence ${(\mu _{k})}_{k\ge 1}$ is exactly chosen in such a way that it satisfies this. Plugging (19) into (17) gives for every $k \ge 1$ the condition

$$\begin{aligned} 1 \le \left( 1 + \frac{1}{t_{k+1}} \right) \frac{t_{k}^2}{t_{k+1}^2}\frac{t_{k+1}}{t_{k+1} - 1} = \frac{t_{k}^2}{t_{k+1}^2} \frac{t_{k+1} +1}{t_{k+1} - 1}, \end{aligned}$$

which is equivalent to

$$\begin{aligned} 0 \ge t_{k+1}^3 - t_{k+1}^2 - t_{k}^2t_{k+1} - t_{k}^2 \end{aligned}$$

and further to

$$\begin{aligned} t_{k+1}^2 + t_{k}^2 \ge t_{k+1}\left( t_{k+1}^2 - t_{k}^2\right) . \end{aligned}$$

Plugging in $t_{k+1} = \sqrt{t_{k}^2+2t_{k}}$ we get that this equivalent to

$$\begin{aligned} t_{k+1}^2 + t_{k}^2 \ge t_{k+1}2t_{k} \quad \forall k \ge 1, \end{aligned}$$

which is evidently fulfilled. Thus, the choices in (16) are indeed feasible for our algorithm.

Now we want to prove the claimed convergence rates. Via induction we show that

20 $$\begin{aligned} \frac{k+1}{2} \le t_{k} \le k \quad \forall k \ge 1. \end{aligned}$$

Evidently, this holds for $t_1=1$. Assuming that (20) holds for $k \ge 1$, we easily see that

$$\begin{aligned} t_{k+1} = \sqrt{t_{k}^2 + 2t_{k}} \le \sqrt{k^2 + 2k} \le \sqrt{k^2 +2k +1} = k+1 \end{aligned}$$

and, on the other hand,

$$\begin{aligned} t_{k+1} = \sqrt{t_{k}^2 + 2t_{k}} \ge \sqrt{\frac{{(k+1)}^2}{4} + k+1} = \frac{1}{2} \sqrt{k^2 + 6k + 5} \ge \frac{1}{2} \sqrt{k^2 + 4k + 4} = \frac{k+2}{2}. \end{aligned}$$

In the following we prove a similar estimate for the sequence ${(\mu _{k})}_{k \ge 1}$. To this end we show, again by induction, the following recursion for every $k\ge 2$

21 $$\begin{aligned} \mu _{k} = \mu _1\frac{\prod _{j=1}^{k-1} t_j}{\prod _{j=2}^{k}(t_j - 1)} \frac{1}{t_{k}}. \end{aligned}$$

For $k=2$ this follows from the definition (19). Assume now that (21) holds for $k \ge 2$. From here we have that

$$\begin{aligned} \mu _{k+1} = \mu _{k} \frac{t_{k}^2}{t_{k+1}(t_{k+1}-1)} = \mu _1\frac{\prod _{j=1}^{k-1} t_j}{\prod _{j=2}^{k}(t_j - 1)} \frac{1}{t_{k}}\frac{t_{k}^2}{t_{k+1}(t_{k+1}-1)} = \mu _1\frac{\prod _{j=1}^{k} t_j}{\prod _{j=2}^{k+1}(t_j - 1)} \frac{1}{t_{k+1}}. \end{aligned}$$

Using (21) together with (20) we can check that for every $k \ge 1$

22 $$\begin{aligned} \begin{aligned} \mu _{k+1} = \mu _1\frac{\prod _{j=1}^{k} t_j}{\prod _{j=2}^{k+1}(t_j - 1)} \frac{1}{t_{k+1}} = \frac{\mu _1}{t_{k+1}} \prod _{j=1}^{k}\frac{t_j}{(t_{j+1} - 1)} \ge \frac{\mu _1}{t_{k+1}} = b \Vert K \Vert ^2\frac{1}{t_{k+1}}, \end{aligned} \end{aligned}$$

where we used in the last step the fact that $t_{k+1} \le t_{k}+1$.

The last thing to check is the fact that $\mu _{k}$ goes to zero like $\frac{1}{k}$. First we check that for every $k\ge 1$

23 $$\begin{aligned} \frac{t_{k}}{t_{k+1} - 1} \le 1 + \frac{1}{t_{k+1}(t_{k+1} - 1)}. \end{aligned}$$

This can be seen via

$$\begin{aligned} (t_{k} + 1) t_{k+1} \le {(t_{k} + 1)}^2 = t_{k+1}^2 + 1 \quad \forall k \ge 1. \end{aligned}$$

By bringing $t_{k+1}$ to the other side we get that

$$\begin{aligned} t_{k+1}t_{k} \le t_{k+1}^2 - t_{k+1} + 1, \end{aligned}$$

from which we can deduce (23) by dividing by $t_{k+1}^2 - t_{k+1}$.

We plug in the estimate (23) in (21) and get for every $k \ge 2$

$$\begin{aligned} \begin{aligned} \mu _{k}&= \mu _1\frac{\prod _{j=1}^{k-1} t_j}{\prod _{j=1}^{k-1}(t_{j+1} - 1)} \frac{1}{t_{k}} \\&\le \mu _1 \prod _{j=1}^{k-1} \left( 1 + \frac{1}{t_{j+1}(t_{j+1} - 1)} \right) \frac{1}{t_{k}} \le \mu _1 \prod _{j=1}^{k-1}\left( 1 + \frac{4}{(j+2)j} \right) \frac{1}{t_{k}} \\&\le \mu _1 \prod _{j=1}^{k-1} \left( 1 + \frac{4}{j^2}\right) \frac{1}{t_{k}} \le \mu _1 \exp \left( \frac{\pi ^2 4}{6}\right) \frac{1}{t_{k}} = b \Vert K \Vert ^2\exp \left( \frac{\pi ^2 4}{6}\right) \frac{1}{t_{k}}. \end{aligned} \end{aligned}$$

With the above inequalities we can to deduce the claimed convergence rates. First note that from Theorem 3.1 we have

$$\begin{aligned} F(x_{N}) - F(x^*) \le \frac{\Vert x_{0} - x^*\Vert ^2 }{2\gamma _{N} t_{N}^{2}} + \mu _{N}\frac{L_{g}^2}{2} \quad \forall N \ge 1. \end{aligned}$$

Now, in order to obtain the desired conclusion, we use the above estimates and deduce for every $N \ge 1$

$$\begin{aligned} \begin{aligned} \frac{\Vert x_{0} - x^*\Vert ^2 }{2\gamma _{N} t_{N}^{2}} + \mu _{N}\frac{L_{g}^2}{2}&\le \frac{\Vert x_{0} - x^*\Vert ^2 }{2 b t_{N}} + \frac{bL_{g}^2\Vert K \Vert ^2}{2 t_N} \exp \left( \frac{4 \pi ^2}{6}\right) \\&\le \frac{\Vert x_{0} - x^*\Vert ^2 }{b (N+1)} + \frac{bL_{g}^2\Vert K \Vert ^2}{(N+1)} \exp \left( \frac{4 \pi ^2}{6}\right) , \end{aligned} \end{aligned}$$

where we used that

$$\begin{aligned} \gamma _N t_N = \frac{\mu _N t_N}{\Vert K \Vert ^2} \ge b, \end{aligned}$$

as shown in (22). $\square$

### Remark 3.3

Consider the choice (see [21])

$$\begin{aligned} t_1 = 1, \quad t_{k+1} = \frac{1 + \sqrt{1 + 4t_{k}^2}}{2} \quad \forall k \ge 1 \end{aligned}$$

and

$$\begin{aligned}\mu _1 = b \Vert K \Vert ^2, \ \text {for} \ b >0.\end{aligned}$$

Since

$$\begin{aligned} t_{k}^2 = t_{k+1}^2 - t_{k+1} \quad \forall k \ge 1, \end{aligned}$$

we see that in this setting we have to choose

$$\begin{aligned} \mu _{k} = b \Vert K \Vert ^2 \ \text {and} \ \gamma _k = b \quad \forall k \ge 1. \end{aligned}$$

Thus, the sequence of optimal function values ${(F(x_N))}_{N \ge 1}$ approaches a $b \Vert K \Vert ^2 \frac{L_g}{2}$-approximation of the optimal objective value $F(x^*)$ with a convergence rate of ${\mathcal {O}}(\frac{1}{N^2})$, i.e.

$$\begin{aligned} F(x_{N}) - F(x^*) \le 2\frac{\Vert x_{0} - x^*\Vert ^2}{b {(N+1)}^2} + b\frac{\Vert K \Vert ^2L_{g}^2}{2} \quad \forall N \ge 1. \end{aligned}$$

## Stochastic Method

### Problem 4.1

The problem is the same as in the deterministic case

$$\begin{aligned} \min _{x \in {\mathcal {H}}{}}\, f(x) + g(Kx) \end{aligned}$$

other than the fact that at each iteration we are only given a stochastic estimator of the quantity

$$\begin{aligned} \nabla ({}^{\mu _{k}}g \circ K)(\cdot ) = K^* {\text {prox}}^{}_{\frac{1}{\mu _{k}}g^*}\left( \frac{1}{\mu _{k}}K\cdot \right) \quad \forall k \ge 1. \end{aligned}$$

### Remark 4.1

See Algorithm 4.3 for a setting where such an estimator is easily computed.

For the stochastic quantities arising in this section we will use the following notation. For every $k \ge 0$, we denote by $\sigma (x_0, \dots , x_k)$ the smallest $\sigma$-algebra generated by the family of random variables $\{x_0, \dots , x_k\}$ and by ${\mathbb {E}}_k(\cdot ) := {\mathbb {E}}(\cdot | \sigma (x_0, \dots , x_k))$ the conditional expectation with respect to this $\sigma$-algebra.

### Algorithm 4.1

(stochastic Variable Accelerated SmooThing (sVAST)) Let $y_0 = x_0 \in {\mathcal {H}}, {(\mu _{k})}_{k \ge 1}$ a sequence of positive and nonincreasing real numbers, and ${(t_{k})}_{k \ge 1}$ a sequence of real numbers with $t_1=1$ and $t_k \ge 1$ for every $k \ge 2$. Consider the following iterative scheme

$$\begin{aligned} (\forall k \ge 1) \quad \left\lfloor \begin{array}{l} L_{k} = \frac{\Vert K \Vert ^2}{\mu _{k}} \\ \gamma _{k} = \frac{1}{L_{k}} \\ x_{k} = {\text {prox}}^{}_{\gamma _{k}f}\left( y_{k-1} - \gamma _{k}\xi _{k-1} \right) \\ y_{k} = x_{k} + \frac{t_{k}-1}{t_{k+1}}(x_{k} - x_{k-1}), \end{array}\right. \end{aligned}$$

where we make the standard assumptions about our gradient estimator of being unbiased, i.e.

$$\begin{aligned} {\mathbb {E}}_{k} (\xi _{k}) = \nabla ({}^{\mu _{k+1}}g \circ K)(y_{k}), \end{aligned}$$

and having bounded variance

$$\begin{aligned} {\mathbb {E}}_{k} \left( \Vert \xi _{k} - \nabla ({}^{\mu _{k+1}}g \circ K)(y_{k}) \Vert ^2 \right) \le \sigma ^2 \end{aligned}$$

for every $$k\ge 0$$.

Note that we use the same notations as in the deterministic case

$$\begin{aligned} u_{k} := x_{k-1} + t_{k}(x_{k} - x_{k-1}) \ \text{ and } \ F^{k}(\cdot ) := f + {}^{\mu _{k}}g\circ K \quad \forall k \ge 1. \end{aligned}$$

### Lemma 4.1

The following statement holds for every (deterministic) $z \in {\mathcal {H}}$ and every $k \ge 0$

$$\begin{aligned} \begin{aligned} {\mathbb {E}}_{k} \left( F^{k+1}(x_{k+1}) + \frac{\Vert x_{k+1} - z \Vert ^2}{2 \gamma _{k+1}}\right) \le&\ F^{k+1}(z) + \frac{\Vert z - y_{k} \Vert ^2}{2 \gamma _{k+1}} \\&\ + {\gamma _{k+1}\left( \sigma ^2 + \frac{\Vert K \Vert ^2L_g^2}{2}\right) } \end{aligned} \end{aligned}$$

### Proof

Here we have to proceed a little bit different from Lemma 3.1. Namely, we have to treat the gradient step and the proximal step differently. For this purpose we define the auxiliary variable

$$\begin{aligned} z_{k} := y_{k-1} - \gamma _{k} \xi _{k-1} \quad \forall k\ge 1. \end{aligned}$$

Let $k \ge 1$ be fixed. From the gradient step we get

$$\begin{aligned} \begin{aligned} \Vert z - z_{k} \Vert ^2&= \Vert y_{k-1} - \gamma _{k}\xi _{k-1} - z \Vert ^2 \\&= \Vert y_{k-1} - z \Vert ^2 - 2\gamma _{k} \left\langle \xi _{k-1}, y_{k-1} - z \right\rangle + \gamma ^2_{k}\Vert \xi _{k-1} \Vert ^2. \end{aligned} \end{aligned}$$

Taking the conditional expectation gives

$$\begin{aligned} \begin{aligned} {\mathbb {E}}_{k-1}\left( \Vert z - z_{k} \Vert ^2\right) \!= \Vert y_{k-1} - z \Vert ^2 \! - 2\gamma _{k} \left\langle \nabla ({}^{\mu _{k}}g \circ K)(y_{k-1}), y_{k-1} - z \right\rangle \! + \gamma ^2_{k}{\mathbb {E}}_{k-1} \left( \Vert \xi _{k-1} \Vert ^2\right) . \end{aligned} \end{aligned}$$

Using the gradient inequality we deduce

$$\begin{aligned} \begin{aligned} {\mathbb {E}}_{k-1}\left( \Vert z - z_{k} \Vert ^2\right) \le&\ \Vert y_{k-1} - z \Vert ^2 - 2\gamma _{k}(({}^{\mu _{k}}g \circ K)(y_{k-1}) - ({}^{\mu _{k}}g \circ K)(z))\\&\ + \gamma ^2_{k}{\mathbb {E}}_{k-1} \left( \Vert \xi _{k-1} \Vert ^2\right) \end{aligned} \end{aligned}$$

and therefore

24 $$\begin{aligned} \begin{aligned} \gamma _{k}({}^{\mu _{k}}g \circ K)(y_{k-1}) + \frac{1}{2}{\mathbb {E}}_{k-1}\left( \Vert z - z_{k} \Vert ^2\right) \le&\ \frac{1}{2}\Vert y_{k-1} - z \Vert ^2 + \gamma _{k}({}^{\mu _{k}}g \circ K)(z) \\&\ + \frac{\gamma ^2_{k}}{2}{\mathbb {E}}_{k-1}\left( \Vert \xi _{k-1} \Vert ^2\right) . \end{aligned} \end{aligned}$$

Also from the smoothness of $({}^{\mu _{k}}g \circ K)$ we deduce via the Descent Lemma that

$$\begin{aligned} \begin{aligned} {}^{\mu _{k}}g (Kz_{k}) \le {}^{\mu _{k}}g (Ky_{k-1}) + \left\langle \nabla ({}^{\mu _{k}}g \circ K)(y_{k-1}), z_{k} - y_{k-1} \right\rangle + \frac{L_{k}}{2} \Vert z_{k} - y_{k-1} \Vert ^2. \end{aligned} \end{aligned}$$

Plugging in the definition of $z_{k}$ and using the fact that $L_{k} = \frac{1}{\gamma _{k}}$ we get

$$\begin{aligned} \begin{aligned} {}^{\mu _{k}}g (Kz_{k}) \le {}^{\mu _{k}}g (Ky_{k-1}) - \gamma _{k} \left\langle \nabla ({}^{\mu _{k}}g \circ K)(y_{k-1}), \xi _{k-1} \right\rangle + \frac{\gamma _{k}}{2} \Vert \xi _{k-1} \Vert ^2. \end{aligned} \end{aligned}$$

Now we take the conditional expectation to deduce that

25 $$\begin{aligned} \begin{aligned} {\mathbb {E}}_{k-1}({}^{\mu _{k}}g (Kz_{k})) \le {}^{\mu _{k}}g (Ky_{k-1}) - \gamma _{k} \left\Vert \nabla ({}^{\mu _{k}}g \circ K)(y_{k-1}) \right\Vert ^2 + \frac{\gamma _{k}}{2} {\mathbb {E}}_{k-1}\left( \Vert \xi _{k-1} \Vert ^2\right) . \end{aligned} \end{aligned}$$

Multiplying (25) by $\gamma _k$ and adding it to (24) gives

$$\begin{aligned}&\gamma _k {\mathbb {E}}_{k-1} \left( {}^{\mu _{k}}g (Kz_k)\right) + \frac{1}{2}{\mathbb {E}}_{k-1}\left( \Vert z - z_k \Vert ^2\right) \\&\quad \le \gamma _k{}^{\mu _{k}}g (Kz) + \frac{1}{2}\Vert y_{k-1} - z \Vert ^2 - \gamma _k^2 \left\Vert \nabla ({}^{\mu _{k}}g \circ K)(y_{k-1}) \right\Vert ^2 + \gamma _k^2 {\mathbb {E}}_{k-1}\left( \Vert \xi _{k-1} \Vert ^2\right) . \end{aligned}$$

Now we use the assumption about the bounded variance to deduce that

26 $$\begin{aligned} \begin{aligned} \gamma _{k}{\mathbb {E}}_{k-1}\left( {}^{\mu _{k}}g (Kz_{k})\right) + \frac{1}{2}{\mathbb {E}}_{k-1}\left( \Vert z - z_{k} \Vert ^2\right) \le \gamma _{k}{}^{\mu _{k}}g (Kz) + \frac{1}{2}\Vert y_{k-1} - z \Vert ^2 + \gamma _{k}^2 \sigma ^2. \end{aligned} \end{aligned}$$

Next up for the proximal step we deduce

27 $$\begin{aligned} f(x_{k}) + \frac{1}{2 \gamma _{k}}\Vert x_{k} - z_{k} \Vert ^2 + \frac{1}{2 \gamma _{k}}\Vert x_{k} - z \Vert ^2 \le f(z) + \frac{1}{2 \gamma _{k}} \Vert z - z_{k} \Vert ^2. \end{aligned}$$

Taking the conditional expectation and combining (26) and (27) we get

$$\begin{aligned} \begin{aligned}&{\mathbb {E}}_{k-1} \left( \gamma _{k}({}^{\mu _{k}}g(Kz_{k}) + f(x_{k})) + \frac{1}{2} \Vert x_{k} - z_{k} \Vert ^2 +\frac{1}{2} \Vert x_{k} - z \Vert ^2 \right) \\&\quad \le \gamma _{k}F^{k}(z) +\frac{1}{2}\Vert y_{k-1} - z \Vert ^2 + \gamma ^2_{k}\sigma ^2. \end{aligned} \end{aligned}$$

From here, using now Lemma 2.3, we get that

$$\begin{aligned} \begin{aligned}&{\mathbb {E}}_{k-1} \left( \gamma _{k}F^{k}(x_{k}) - \gamma _{k}L_g\Vert K \Vert \Vert x_{k} - z_{k} \Vert + \frac{1}{2} \Vert x_k - z_k \Vert ^2 +\frac{1}{2} \Vert x_{k} - z \Vert ^2 \right) \\&\quad \le \gamma _{k}F^{k}(z) +\frac{1}{2}\Vert y_{k-1} - z \Vert ^2 + \gamma ^2_{k}\sigma ^2. \end{aligned} \end{aligned}$$

Now we use

$$\begin{aligned} {- \frac{1}{2}\gamma _k^2 L_g^2 \Vert K \Vert ^2 \le \frac{1}{2}\Vert x_k - z_k \Vert ^2 - \gamma _k L_g\Vert K \Vert \Vert x_k - z_k \Vert } \end{aligned}$$

to obtain that

$$\begin{aligned} \begin{aligned}&{\mathbb {E}}_{k-1} \left( \gamma _{k}F^{k}(x_{k}) + \frac{1}{2} \Vert x_{k} - z \Vert ^2 \right) \\&\quad \le \gamma _{k}F^{k}(z) +\frac{1}{2}\Vert y_{k-1} - z \Vert ^2 + \gamma ^2_{k}\sigma ^2 + {\frac{1}{2} \gamma _k^2 L_g^2 \Vert K \Vert ^2.} \end{aligned} \end{aligned}$$

$$\square $$

### Lemma 4.2

Let $x^*$ be an optimal solution of Problem 4.1. Then it holds

$$\begin{aligned} \begin{aligned} {\mathbb {E}}\left( \gamma _{1}(F^{1}(x_{1}) - F^{1}(x^*))\right) + \frac{1}{2} \Vert u_{1} - x^* \Vert ^2 \le \frac{1}{2}\Vert x_{0} - x^* \Vert ^2 + \gamma ^2_{1}\sigma ^2 + {\frac{1}{2} \gamma _1^2 L_g^2 \Vert K \Vert ^2.} \end{aligned} \end{aligned}$$

### Proof

Applying the previous lemma with $k=0$ and $z = x^*$, we get that

$$\begin{aligned} \begin{aligned} {\mathbb {E}}\left( \gamma _{1}F^{1}(x_{1}) + \frac{1}{2} \Vert x_{1} - x^* \Vert ^2\right) \! \le \! \gamma _{1}F^{1}(x^*) +\frac{1}{2}\Vert y_{0} - x^* \Vert ^2 + \gamma ^2_{1}\sigma ^2 + {\frac{1}{2} \gamma _1^2 L_g^2\Vert K \Vert ^2.} \end{aligned} \end{aligned}$$

Therefore, using the fact that $y_{0} = x_{0}$ and $u_{1} = x_{1}$,

$$\begin{aligned} \begin{aligned} {\mathbb {E}}\left( \gamma _{1}(F^{1}(x_{1}) - F^{1}(x^*)) + \frac{1}{2} \Vert u_{1} - x^* \Vert ^2\right) \le \frac{1}{2}\Vert x_{0} - x^* \Vert ^2 + \gamma ^2_{1}\sigma ^2 + {\frac{1}{2} \gamma _1^2 L_g^2 \Vert K \Vert ^2}, \end{aligned} \end{aligned}$$

which finishes the proof. $\square$

### Theorem 4.1

Consider the setup of Problem 4.1 and let ${(x_{k})}_{k \ge 0}$ and ${(y_{k})}_{k \ge 0}$ denote the sequences generated by Algorithm 4.1. Assume that for all $k\ge 1$

$$\begin{aligned} \rho _{k+1} := t_{k}^2 - t_{k+1}^2 + t_{k+1} \ge 0. \end{aligned}$$

Then, for every optimal solution $x^*$ of Problem 4.1, it holds

$$\begin{aligned} {\mathbb {E}}\left( F(x_{N}) - F(x^*)\right) \le&\ \frac{1}{\gamma _N t_N^2} \frac{1}{2}\Vert x_0 - x^*\Vert ^2 + {\frac{1}{\gamma _N t_N^2}\frac{\Vert K \Vert ^2 L_g^2}{2}\sum _{k=1}^N \gamma _k^2(t_k + \rho _k)} \\&\ + {\frac{1}{\gamma _N t_N^2} \left( \sigma ^2 + \frac{\Vert K \Vert ^2 L_g^2}{2} \right) \sum _{k=1}^N t_k^2\gamma _k^2} \quad \forall N \ge 1. \end{aligned}$$

### Proof of Theorem 4.1

Let $k \ge 0$ be fixed. Lemma 4.1 for $z := \left( 1- \frac{1}{t_{k+1}} \right) x_{k} + \frac{1}{t_{k+1}} x^*$ gives

$$\begin{aligned}&{\mathbb {E}}_{k} \left( F^{k+1}(x_{k+1}) + \frac{1}{2 \gamma _{k+1}}\left\Vert \frac{1}{t_{k+1}} u_{k+1} - \frac{1}{t_{k+1}} x^* \right\Vert ^2 \right) \\&\quad \le F^{k+1}\left( \left( 1- \frac{1}{t_{k+1}} \right) x_{k} + \frac{1}{t_{k+1}} x^*\right) + \frac{1}{2 \gamma _{k+1}}\left\Vert \frac{1}{t_{k+1}} x^* - \frac{1}{t_{k+1}} u_{k} \right\Vert ^2 \\&\quad +{\gamma _{k+1}\left( \sigma ^2 + \frac{\Vert K \Vert ^2L_g^2}{2}\right) }. \end{aligned}$$

From here and from the convexity of $F^{k+1}$ follows

$$\begin{aligned}&{\mathbb {E}}_k \left( F^{k+1}(x_{k+1}) - F^{k+1}(x^*) \right) - \left( 1 - \frac{1}{t_{k+1}} \right) (F^{k+1}(x_{k}) - F^{k+1}(x^*)) \\&\quad \le \frac{\Vert u_{k} - x^* \Vert ^2}{2 \gamma _{k+1} t_{k+1}^2} - {\mathbb {E}}_{k}\left( \frac{\Vert u_{k+1} - x^* \Vert ^2}{2 \gamma _{k+1} t_{k+1}^2}\right) + {\gamma _{k+1}\left( \sigma ^2 + \frac{\Vert K \Vert ^2 L_g^2}{2}\right) }. \end{aligned}$$

Now, by multiplying both sides with by $t_{k+1}^2$, we deduce

28 $$\begin{aligned} \begin{aligned}&{\mathbb {E}}_k \left( t_{k+1}^2 (F^{k+1}(x_{k+1}) - F^{k+1}(x^*)) \right) + (t_{k+1} - t_{k+1}^2)(F^{k+1}(x_k) - F^{k+1}(x^*)) \\&\quad \le \frac{1}{2 \gamma _{k+1}} \left( \Vert u_{k} - x^* \Vert ^2 - {\mathbb {E}}_{k}\left( \Vert u_{k+1} - x^* \Vert ^2 \right) \right) +{t_{k+1}^2\gamma _{k+1}\left( \sigma ^2 + \frac{\Vert K \Vert ^2 L_g^2}{2}\right) }. \end{aligned} \end{aligned}$$

Next, by adding $t_{k}^2(F^{k+1}(x_{k}) - F^{k+1}(x^*))$ on both sides of (28), gives

$$\begin{aligned}&{\mathbb {E}}_{k} \left( t_{k+1}^2 (F^{k+1}(x_{k+1}) - F^{k+1}(x^*))\right) + \rho _{k+1}(F^{k+1}(x_{k}) - F^{k+1}(x^*)) \le \\&\quad t_{k}^2(F^{k+1}(x_{k}) - F^{k+1}(x^*)) + \frac{1}{2 \gamma _{k+1}} \left( \Vert u_{k} - x^* \Vert ^2 - {\mathbb {E}}_{k} \left( \Vert u_{k+1} - x^* \Vert ^2 \right) \right) \\&\quad + {t_{k+1}^2\gamma _{k+1}\left( \sigma ^2 + \frac{\Vert K \Vert ^2L_g^2}{2}\right) }. \end{aligned}$$

Utilizing (6) together with the assumption that ${(\mu _{k})}_{k \ge 1}$ is nonincreasing leads to

$$\begin{aligned}&{\mathbb {E}}_{k} \left( t_{k+1}^2 (F^{k+1}(x_{k+1}) - F^{k+1}(x^*)) \right) + \rho _{k+1}(F^{k+1}(x_{k}) - F^{k+1}(x^*)) \\&\quad \le t_{k}^2(F^{k}(x_{k}) - F^{k}(x^*)) + \frac{1}{2\gamma _{k+1}} \left( \Vert u_{k} - x^* \Vert ^2 - {\mathbb {E}}_{k} \left( \Vert u_{k+1} - x^* \Vert ^2 \right) \right) + t_{k}^2(\mu _{k} - \mu _{k+1})\frac{L_{g}^2}{2} \\&\quad + {t_{k+1}^2\gamma _{k+1}\left( \sigma ^2 + \frac{\Vert K \Vert ^2 L_g^2}{2}\right) }. \end{aligned}$$

Now, using that $t_{k}^2 \ge t_{k+1}^2 - t_{k+1}$, we get

$$\begin{aligned}&{\mathbb {E}}_{k}\left( t_{k+1}^2 (F^{k+1}(x_{k+1}) - F^{k+1}(x^*)) \right) + \rho _{k+1}(F^{k+1}(x_{k}) - F^{k+1}(x^*)) \\&\quad \le t_{k}^2(F^{k}(x_{k}) - F^{k}(x^*)) + \frac{1}{2\gamma _{k+1}} (\Vert u_{k} - x^* \Vert ^2 - {\mathbb {E}}_{k}\left( \Vert u_{k+1} - x^* \Vert ^2) \right) \\&\quad + t_{k}^2\mu _{k} \frac{L_{g}^2}{2} - t_{k+1}^2 \mu _{k+1}\frac{L_{g}^2}{2} + t_{k+1} \mu _{k+1}\frac{L_{g}^2}{2} \\&\quad + {t_{k+1}^2\gamma _{k+1}\left( \sigma ^2 + \frac{\Vert K \Vert ^2 L_g^2}{2}\right) }. \end{aligned}$$

Multiplying both sides with $\gamma _{k+1}$ and putting all terms on the correct sides yields

29 $$\begin{aligned} \begin{aligned}&{\mathbb {E}}_{k}\Bigg (\gamma _{k+1}t_{k+1}^2 \left( F^{k+1}(x_{k+1}) - F^{k+1}(x^*) + \mu _{k+1}\frac{L_{g}^2}{2}\right) + \frac{1}{2}\Vert u_{k+1} - x^* \Vert ^2 \Bigg ) + \\&\quad \gamma _{k+1}\rho _{k+1}(F^{k+1}(x_{k}) - F^{k+1}(x^*)) \\&\quad \le \gamma _{k+1}t_{k}^2\left( F^{k}(x_{k}) - F^{k}(x^*) + \mu _{k}\frac{L_{g}^2}{2}\right) + \frac{1}{2}\Vert u_{k} - x^* \Vert ^2 \\&\quad + \gamma _{k+1} t_{k+1} \mu _{k+1}\frac{L_{g}^2}{2} + {t_{k+1}^2\gamma _{k+1}^2\left( \sigma ^2 + \frac{\Vert K \Vert ^2L_g^2}{2}\right) }.&\end{aligned} \end{aligned}$$

At this point we would like to discard the term $\gamma _{k+1} \rho _{k+1}(F^{k+1}(x_{k}) - F^{k+1}(x^*))$ which we currently cannot as the positivity of $F^{k+1}(x_{k}) - F^{k+1}(x^*)$ is not ensured. So we add $\gamma _{k+1}\rho _{k+1}\mu _{k+1} \frac{L_{g}^2}{2}$ on both sides of (29) and get

30 $$\begin{aligned} \begin{aligned}&{\mathbb {E}}_k\Bigg (\gamma _{k+1}t_{k+1}^2 \left( F^{k+1}(x_{k+1}) - F^{k+1}(x^*) + \mu _{k+1}\frac{L_{g}^2}{2}\right) + \frac{1}{2}\Vert u_{k+1} - x^* \Vert ^2 \Bigg ) + \\&\quad \gamma _{k+1}\rho _{k+1}\left( F^{k+1}(x_{k}) - F^{k+1}(x^*) + \mu _{k+1}\frac{L_{g}^2}{2}\right) \le \\&\quad \gamma _{k+1}t_{k}^2\left( F^{k}(x_{k}) - F^{k}(x^*) + \mu _{k}\frac{L_{g}^2}{2} \right) + \frac{1}{2}\Vert u_{k} - x^* \Vert ^2 + \\&\quad + \gamma _{k+1} \mu _{k+1}\frac{L_{g}^2}{2}(t_{k+1}+\rho _{k+1}) +{t_{k+1}^2\gamma _{k+1}^2\left( \sigma ^2 + \frac{\Vert K \Vert ^2 L_g^2}{2}\right) }. \end{aligned} \end{aligned}$$

Using again (6) to deduce that

$$\begin{aligned} \gamma _{k+1}\rho _{k+1}\left( F^{k+1}(x_{k}) - F^{k+1}(x^*) + \mu _{k+1}\frac{L_{g}^2}{2}\right) \ge \gamma _{k+1}\rho _{k+1}(F(x_{k}) - F(x^*)) \ge 0 \end{aligned}$$

we can now discard said term from (30), giving

31 $$\begin{aligned} \begin{aligned}&{\mathbb {E}}_{k}\left( \gamma _{k+1}t_{k+1}^2 \left( F^{k+1}(x_{k+1}) - F^{k+1}(x^*) + \mu _{k+1}\frac{L_{g}^2}{2}\right) + \frac{1}{2}\Vert u_{k+1} - x^* \Vert ^2 \right) \le \\&\quad \gamma _{k+1}t_{k}^2\left( F^{k}(x_{k}) - F^{k}(x^*) + \mu _{k}\frac{L_{g}^2}{2}\right) + \frac{1}{2}\Vert u_{k} - x^* \Vert ^2 \\&\quad + \gamma _{k+1} \mu _{k+1}\frac{L_{g}^2}{2}(t_{k+1}+\rho _{k+1}) +{t_{k+1}^2\gamma _{k+1}^2\left( \sigma ^2 + \frac{\Vert K \Vert ^2 L_g^2}{2}\right) }. \end{aligned} \end{aligned}$$

Last but not least we use that $F^{k}(x_{k}) - F^{k}(x^*) + \mu _{k}\frac{L_{g}^2}{2} \ge F(x_{k})-F(x^*)\ge 0$ and $\gamma _{k+1} \le \gamma _{k}$ to follow that

32 $$\begin{aligned} \gamma _{k+1}t_{k}^2\left( F^{k}(x_{k}) - F^{k}(x^*) + \mu _{k}\frac{L_{g}^2}{2}\right) \le \gamma _{k}t_{k}^2\left( F^{k}(x_{k}) - F^{k}(x^*) + \mu _{k}\frac{L_{g}^2}{2}\right) . \end{aligned}$$

Combining (31) and (32) yields

33 $$\begin{aligned} \begin{aligned}&{\mathbb {E}}_{k}\left( \gamma _{k+1}t_{k+1}^2 \left( F^{k+1}(x_{k+1}) - F^{k+1}(x^*) + \mu _{k+1}\frac{L_{g}^2}{2}\right) + \frac{1}{2}\Vert u_{k+1} - x^* \Vert ^2 \right) \le \\&\quad \gamma _{k}t_{k}^2\left( F^{k}(x_{k}) - F^{k}(x^*) + \mu _{k}\frac{L_{g}^2}{2}\right) + \frac{1}{2}\Vert u_{k} - x^* \Vert ^2 \\&\quad + \gamma _{k+1} \mu _{k+1}\frac{L_{g}^2}{2}(t_{k+1}+\rho _{k+1}) +{t_{k+1}^2\gamma _{k+1}^2\left( \sigma ^2 + \frac{\Vert K \Vert ^2 L_g^2}{2}\right) }. \end{aligned} \end{aligned}$$

Let $N \ge 2$. We take the expected value on both sides (33) and sum from $k=1$ to $N-1$. Getting rid of the non-negative terms $\Vert u_{N} - x^* \Vert ^2$ gives

$$\begin{aligned} \begin{aligned}&{\mathbb {E}}\left( \gamma _N t_{N}^{2}\left( F^{N}(x_{N}) - F^{N}(x^*) + \mu _{N}\frac{L_{g}^2}{2}\right) \right) \le \\&\quad {\mathbb {E}}\left( \gamma _{1}\left( F^{1}(x_{1}) - F^{1}(x^*) + \mu _{1}\frac{L_g^2}{2}\right) \right) + \frac{1}{2}\Vert u_{1}- x^* \Vert ^2 + \sum _{k=2}^{N} \gamma _{k} \mu _{k}\frac{L_{g}}{2}(t_{k} + \rho _{k}) \\&\quad + {\sum _{k=2}^{N} t_{k}^2\gamma _{k}^2\left( \sigma ^2 + \frac{\Vert K \Vert ^2 L_g^2}{2}\right) }. \end{aligned} \end{aligned}$$

Since $t_1=1$, the above inequality holds also for $N=1$. Now, using Lemma 4.2 we get that for every $N \ge 1$

$$\begin{aligned}&{\mathbb {E}}\left( \gamma _{N} t_{N}^{2}\left( F^{N}(x_{N}) - F^{N}(x^*) + \mu _{N}\frac{L_{g}^2}{2} \right) \right) \le \ \frac{1}{2}\Vert x_{0} - x^*\Vert ^2 + \sum _{k=1}^{N} \gamma _{k} \mu _{k}\frac{L_g^2}{2}(t_{k} + \rho _{k}) \\&\quad \ + {\sum _{k=1}^{N} t_{k}^2\gamma _{k}^2\left( \sigma ^2 + \frac{\Vert K \Vert ^2 L_g^2}{2}\right) }. \end{aligned}$$

From (7) we follow that

$$\begin{aligned} \begin{aligned}&\gamma _{N} t_{N}^{2} \left( F(x_{N}) - F(x^*) \right) \le \gamma _{N} t_{N}^{2} \left( F^{N}(x_{N}) - F^{N}(x^*) + \mu _{N}\frac{L_g^2}{2} \right) , \end{aligned} \end{aligned}$$

therefore, for every $N \ge 1$

$$\begin{aligned}&{\mathbb {E}}\left( \gamma _{N} t_{N}^{2}\left( F^{N}(x_{N}) - F^{N}(x^*) \right) \right) \le \ \frac{1}{2}\Vert x_{0} - x^*\Vert ^2 + \sum _{k=1}^{N} \gamma _{k} \mu _{k}\frac{L_g^2}{2}(t_{k} + \rho _{k}) \\&\quad \ + {\sum _{k=1}^{N} t_{k}^2\gamma _{k}^2\left( \sigma ^2 + \frac{\Vert K \Vert ^2 L_g^2}{2}\right) }. \end{aligned}$$

By using the fact that $\mu _k = \gamma _k \Vert K \Vert ^2$ for every $k \ge 1$ gives

$$\begin{aligned}&{\mathbb {E}}\left( \gamma _{N} t_{N}^{2}(F(x_{N}) - F(x^*)) \right) \le \ \frac{1}{2}\Vert x_0 - x^*\Vert ^2 + \frac{\Vert K \Vert ^2 L_g^2}{2} \sum _{k=1}^N \gamma _k^2(t_{k} + \rho _k) \\&\quad \ + {\left( \sigma ^2 + \frac{\Vert K \Vert ^2 L_g^2}{2} \right) \sum _{k=1}^{N} t_k^2\gamma _k^2} \quad \forall N \ge 1. \end{aligned}$$

Thus,

$$\begin{aligned}&{\mathbb {E}}\left( F(x_N) - F(x^*) \right) \le \ \frac{1}{\gamma _N t_N^2} \frac{1}{2}\Vert x_0 - x^*\Vert ^2 + \frac{1}{\gamma _N t_N^2}\frac{\Vert K \Vert ^2 L_g^2}{2}\sum _{k=1}^N \gamma _k^2(t_k + \rho _k) \\&\quad \ + {\frac{1}{\gamma _N t_N^2} \left( \sigma ^2 + \frac{\Vert K \Vert ^2 L_g^2}{2} \right) \sum _{k=1}^N t_k^2\gamma _k^2} \quad \forall N \ge 1. \end{aligned}$$

$$\square $$

### Corollary 4.1

Let

$$\begin{aligned} t_1=1, \quad t_{k+1} = \frac{1 + \sqrt{1 + 4 t_{k}^2}}{2} \quad \forall k \ge 1, \end{aligned}$$

and, for $$b >0$$,

$$\begin{aligned} \mu _{k} = \frac{b}{k^{\frac{3}{2}}} \Vert K \Vert ^2, \ \text {and} \ \gamma _k = \frac{b}{k^{\frac{3}{2}}} \quad \forall k \ge 1. \end{aligned}$$

Then,

$$\begin{aligned}&{\mathbb {E}}\left( F(x_N) - F(x^*) \right) \le \ 2\frac{\Vert x_0 - x^* \Vert ^2}{b\sqrt{N}} + b \Vert K \Vert ^2 L_g^2 \frac{\pi ^2}{3}\frac{1}{\sqrt{N}} \\&\quad \ + 2b\left( 2\sigma ^2 + \Vert K \Vert ^2L_g^2 \right) \frac{1+\log (N)}{\sqrt{N}} \quad \forall N \ge 1. \end{aligned}$$

Furthermore, we have that $F(x_N)$ converges almost surely to $F(x^*)$ as $N \rightarrow +\infty$.

### Proof

First we notice that the choice of $t_{k+1} = \frac{1 + \sqrt{1 + 4 t_{k}^2}}{2}$ fulfills that

$$\begin{aligned} \rho _{k+1} = t_{k}^2 - t_{k+1}^2 + t_{k+1} = 0 \quad \forall k \ge 1. \end{aligned}$$

Now we derive the stated convergence result by first showing via induction that

$$\begin{aligned} \frac{1}{k} \le \frac{1}{t_{k}} \le \frac{2}{k} \quad \forall k \ge 1. \end{aligned}$$

Assuming that this holds for $k \ge 1$, we have that

$$\begin{aligned} t_{k+1} = \frac{1+\sqrt{1+4t_{k}^2}}{2} \le \frac{1+\sqrt{1+4k^2}}{2} \le \frac{1+\sqrt{1+4k+4k^2}}{2} = k+1 \end{aligned}$$

and

$$\begin{aligned} t_{k+1} =\frac{1+\sqrt{1+4t_{k}^2}}{2} \ge \frac{1+\sqrt{1+4{(\frac{k}{2})}^2}}{2} \ge \frac{1+\sqrt{k^2}}{2}\ge \frac{k+1}{2}. \end{aligned}$$

Furthermore, for every $N \ge 1$ we have that

34 $$\begin{aligned} \begin{aligned}&\frac{1}{\gamma _N t_N^{2}}\frac{\Vert K \Vert ^2 L_g^2}{2}\sum _{k=1}^N \gamma _k^2(t_k + \rho _k) \le \ \frac{4}{b\sqrt{N}}\frac{\Vert K \Vert ^2 L_g^2}{2}\sum _{k=1}^N \frac{b^2}{k^3}k = \frac{2b \Vert K \Vert ^2 L_g^2}{\sqrt{N}}\sum _{k=1}^N k^{-2} \\&\quad \le \ \frac{2b \Vert K \Vert ^2 L_g^2}{\sqrt{N}}\sum _{k=1}^\infty k^{-2} = b \Vert K \Vert ^2 L_g^2 \frac{\pi ^2}{3}\frac{1}{\sqrt{N}}. \end{aligned} \end{aligned}$$

The statement of the convergence rate in expectation follows now by plugging in our parameter choices into the statement of Theorem 4.1, using the estimate (34) and checking that

$$\begin{aligned} \sum _{k=1}^N t_k^2 \gamma _k^2 \le b^2 \sum _{k=1}^N \frac{1}{k} \le b^2 (1+\log (N)) \quad \forall N \ge 1. \end{aligned}$$

The almost sure convergence of ${(F(x_N))}_{N \ge 1}$ can be deduced by looking at (33) and dividing by $\gamma _{k+1}t_{k+1}^2$ and using that $\gamma _{k+1}t_{k+1}^2 \ge \gamma _{k}t_{k}^2$ as well as $\rho _k=0$, which gives for every $k \ge 0$

$$\begin{aligned} \begin{aligned}&{\mathbb {E}}_k\left( F^{k+1}(x_{k+1}) - F^{k+1}(x^*) + \mu _{k+1}\frac{L_{g}^2}{2} + \frac{1}{2\gamma _{k+1}t_{k+1}^2 }\Vert u_{k+1} - x^* \Vert ^2 \right) \\&\quad \le F^k(x_{k}) - F^{k}(x^*) + \mu _{k}\frac{L_{g}^2}{2} + \! \frac{1}{2\gamma _kt_k^2 }\Vert u_{k} - x^* \Vert ^2 + \! \frac{\mu _{k+1}}{t_{k+1}}\frac{L_{g}^2}{2} +\gamma _{k+1}\left( \sigma ^2 + \frac{\Vert K \Vert ^2 L_g^2}{2} \right) . \end{aligned} \end{aligned}$$

Plugging in our choice of parameters gives for every $k \ge 0$

$$\begin{aligned} \begin{aligned}&{\mathbb {E}}_{k}\left( F^{k+1}(x_{k+1}) - F^{k+1}(x^*) + \mu _{k+1}\frac{L_{g}^2}{2} + \frac{1}{2\gamma _{k+1}t_{k+1}^2 }\Vert u_{k+1} - x^* \Vert ^2 \right) \\&\quad \le F^{k}(x_{k}) - F^{k}(x^*) + \mu _{k}\frac{L_{g}^2}{2} + \frac{1}{2\gamma _{k}t_{k}^2 }\Vert u_{k} - x^* \Vert ^2 +\frac{C}{k^{\frac{3}{2}}}, \end{aligned} \end{aligned}$$

where $$C >0$$.

Thus, by the famous Robbins-Siegmund Theorem (see [25, Theorem 1]) we get that ${(F^{k+1}(x_{k+1}) - F^{k+1}(x^*) + \mu _{k+1}\frac{L_{g}^2}{2})}_{k \ge 0}$ converges almost surely. In particular, from the convergence to 0 in expectation we know that the almost sure limit must also be the constant zero. $\square$

**Finite Sum** The formulation of the previous section can be used to deal e.g. with problems of the form

35 $$\begin{aligned} \min _{x \in {\mathcal {H}}}\, f(x) + \sum _{i=1}^{m} g_{i}(K_{i}x) \end{aligned}$$

for $f:{\mathcal {H}}\rightarrow {\overline{{\mathbb {R}}}}$ a proper, convex and lower semicontinuous function, $g_{i}:{\mathcal {G}}_{i} \rightarrow {\mathbb {R}}$ convex and $L_{g_i}$-Lipschitz continuous functions and $K_{i}: {\mathcal {H}}\rightarrow {\mathcal {G}}_{i}$ linear continuous operators for $i=1,\dots ,m$.

Clearly one could considerwith $\Vert {\varvec{K}} \Vert ^2 = \sum _{i=1}^{m}\Vert K_{i} \Vert ^2$ andin order to reformulate the problem as

$$\begin{aligned} \min _{x \in {\mathcal {H}}}\, f(x) + {\varvec{g}}({\varvec{K}}x) \end{aligned}$$

and use Algorithm 3.1 together with the parameter choices described in Corollary 3.1 on this. This results in the following algorithm.

### Algorithm 4.2

Let $$y_0 = x_0 \in {\mathcal {H}}, \mu _{1}=b \Vert {\varvec{K}} \Vert $$, for $$b >0 $$, and $$t_1=1$$. Consider the following iterative scheme

$$\begin{aligned} (\forall k \ge 1) \quad \left\lfloor \begin{array}{l} \gamma _{k} = \frac{\sum _{i=1}^{m} \Vert K_i \Vert ^2}{\mu _{k}} \\ x_{k} = {\text {prox}}^{}_{\gamma _{k}f}\left( y_{k-1} - \gamma _{k} \sum _{i=1}^m K^*_i{\text {prox}}^{}_{\frac{1}{\mu _{k}}g^*_i}\left( \frac{K_i y_{k-1}}{\mu _{k}} \right) \right) \\ t_{k+1} = \sqrt{t_{k}^2 + 2t_{k}} \\ y_{k} = x_{k} + \frac{t_{k}-1}{t_{k+1}}(x_{k} - x_{k-1}) \\ \mu _{k+1} = \mu _{k} \frac{t_{k}^2}{t_{k+1}^2 - t_{k+1}}. \end{array}\right. \end{aligned}$$

However, Problem (35) also lends itself to be tackled via the stochastic version of our method, Algorithm 4.1, by randomly choosing a subset of the summands. Together with the parameter choices described in Corollary 4.1 which results in the following scheme.

### Algorithm 4.3

Let $y_0 = x_0 \in {\mathcal {H}}, b >0$, and $t_1=1$. Consider the following iterative scheme

$$\begin{aligned} (\forall k \ge 1) \quad \left\lfloor \begin{array}{l} \mu _{k} = b\sum _{i=1}^{m} \Vert K_i \Vert ^2 k^{-\frac{3}{2}} \\ \gamma _{k} = b k^{-\frac{3}{2}} \\ x_{k} = {\text {prox}}^{}_{\gamma _{k} f}\left( y_{k-1} - \gamma _{k} \frac{\epsilon _{i,k}}{p_i} \sum _{i=1}^m K^*_i{\text {prox}}^{}_{\frac{1}{\mu _{k}}g^*_i}\left( \frac{K_i y_{k-1}}{\mu _{k}} \right) \right) \\ t_{k+1} = \frac{1 + \sqrt{1 + 4 t_{k}^2}}{2} \\ y_{k} = x_{k} + \frac{t_{k}-1}{t_{k+1}}(x_{k} - x_{k-1}), \end{array}\right. \end{aligned}$$

with $\epsilon _{k} := (\epsilon _{1,k}, \epsilon _{2,k}, \dots , \epsilon _{m,k})$ a sequence of i.i.d., ${\{0,1\}}^m$ random variables and $p_i = {\mathbb {P}}[\epsilon _{i,1} = 1]$.

Since the above two methods were not explicitly developed for this separable case and can therefore not make use of more refined estimation of the constant $\Vert {\varvec{K}} \Vert$, as it is done in e.g. [14]. However, in the stochastic case, this fact is remedied due to the scaling of the stepsize with respect to the *i*-th component by $p_{i}^{-1}$.

### Remark 4.2

In theory Algorithm 4.1 could be used to treat more general stochastic problems than finite sums like (35), but in the former case it is not clear anymore how a gradient estimator can be found, so we do not discuss it here.

## Numerical Examples

We will focus our numerical experiments on image processing problems. The examples are implemented in python using the operator discretization library (ODL) [1]. We define the discrete gradient operators $D_1$ and $D_2$ representing the discretized derivative in the first and second coordinate respectively, which we will need for the numerical examples. Both map from ${\mathbb {R}}^{m \times n}$ to ${\mathbb {R}}^{m \times n}$ and are defined by

$$\begin{aligned} {(D_1 u)}_{i,j} := {\left\{ \begin{array}{ll} u_{i+1,j} - u_{i,j} &{} 1 \le i < m, \\ 0 &{} \text {else,} \end{array}\right. } \end{aligned}$$

and

$$\begin{aligned} {(D_2 u)}_{i,j} := {\left\{ \begin{array}{ll} u_{i,j+1} - u_{i,j} &{} 1 \le j < m, \\ 0 &{} \text {else.} \end{array}\right. } \end{aligned}$$

The operator norm of $D_1$ and $D_2$, respectively, is 2 (where we equipped ${\mathbb {R}}^{m \times n}$ with the Frobenius norm). This yields an operator norm of $\sqrt{8}$ for the total gradient $D := D_1 \times D_2$ as a map from ${\mathbb {R}}^{m \times n}$ to ${\mathbb {R}}^{m \times n} \times {\mathbb {R}}^{m \times n}$, see also [12].

We will compare our methods, i.e. the Variable Accelerated SmooThing (VAST) and its stochastic counterpart (sVAST) to the Primal Dual Hybrid Gradient (PDHG) of [15] as well as its stochastic version (sPDHG) from [14]. Furthermore, we will illustrate another competitor, the method by Pesquet and Repetti, see [24], which is another stochastic version of PDHG (see also [29]).

In all examples we choose the parameters in accordance with [14]:

- for PDHG and Pesquet&Repetti: $\tau = \sigma _i = \frac{\gamma }{\Vert K \Vert }$
- for sPDHG: $\sigma _i = \frac{\gamma }{\Vert K \Vert }$ and $\tau = \frac{\gamma }{n \max _i \Vert K_i \Vert }$,

where $$\gamma = 0.99$$.

### Total Variation Denoising

The task at hand is to reconstruct an image from its noisy observation. We do this by solving

$$\begin{aligned} \min _{x \in {\mathbb {R}}^{m \times n}{}}\, \alpha \Vert x - b \Vert _2 + \Vert D_1 x \Vert _1 + \Vert D_2 x \Vert _1, \end{aligned}$$

with $\alpha >0$ as regularization parameter, in the following setting: $f= \alpha \Vert \cdot - b \Vert _2, g_1=g_2 = \Vert \cdot \Vert _1, K_1=D_1, K_2=D_2$.

**Fig. 1:**
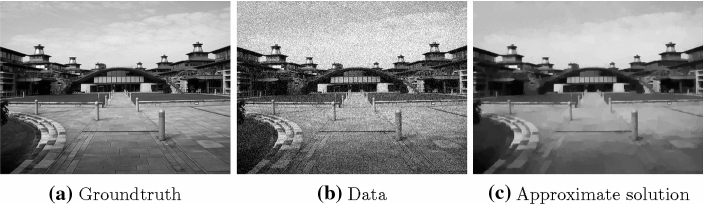
TV denoising. Images used. The approximate solution is computed by running PDHG for 7000 iterations

**Fig. 2:**
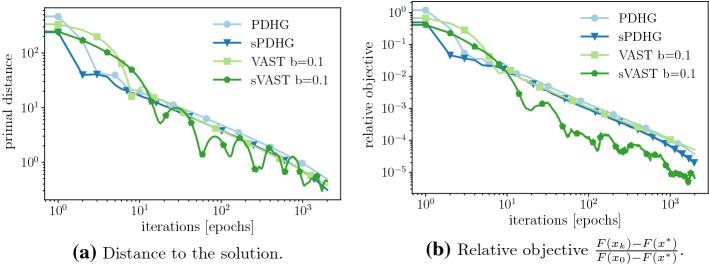
TV denoising. Plots illustrating the performance of different methods

Figure 1 illustrates the images (of dimension $m = 442$ and $n = 331$) used in for this example. These include the groundtruth, i.e. the uncorrupted image, as well as the data for the optimization problem *b*, which visualizes the level of noise. In Fig. 2 we can see that for the deterministic setting our method is as good as PDHG. For the objective function values, Fig. 2b, this is not too surprising as both algorithms share the same convergence rate. For the distance to a solution however we completely lack a convergence result. Nevertheless in Fig. 2a we can see that our method performs also well with respect to this measure.

In the stochastic setting we can see in Fig. 2 that, while sPDHG provides some benefit over its deterministic counterpart, the stochastic version of our method, although significantly increasing the variance, provides great benefit, at least for the objective function values.

**Fig. 3:**
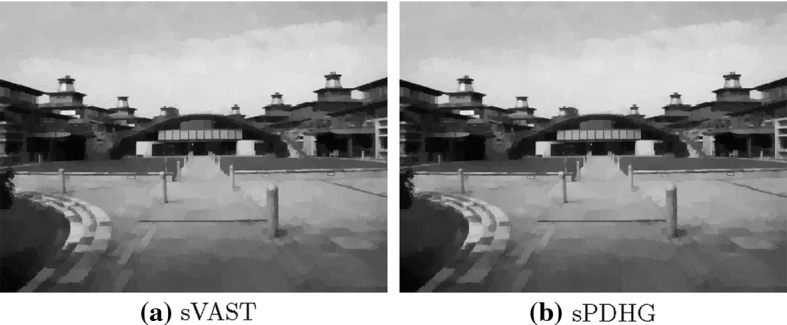
TV Denoising. A comparison of the reconstruction for the stochastic variable smoothing method and the stochastic PDHG

Furthermore, Fig. 3, shows the reconstructions of sPDHG and our method which are, despite the different objective function values, quite comparable.

### Total Variation Deblurring

For this example we want to reconstruct an image from a blurred and noisy image. We assume to know the blurring operator $C: {\mathbb {R}}^{m \times n} \rightarrow {\mathbb {R}}^{m \times n}$. This is done by solving

36 $$\begin{aligned} \min _{x \in {\mathbb {R}}^{m \times n}{}}\, \alpha \Vert C x - b \Vert _2 + \Vert D_1 x \Vert _1 + \Vert D_2 x \Vert _1, \end{aligned}$$

for $\alpha >0$ as regularization parameter, in the following setting: $f=0, g_1 = \alpha \Vert \cdot - b \Vert _2, g_2=g_3 = \Vert \cdot \Vert _1, K_1=C, K_2 = D_1, K_2=D_2$.

**Fig. 4:**
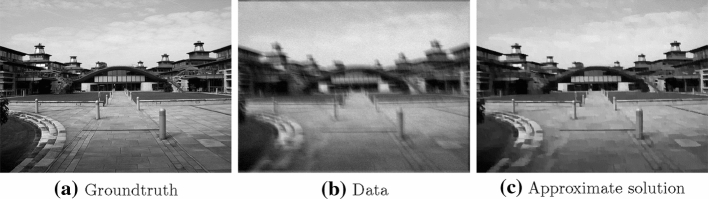
TV Deblurring. The approximate solution is computed by running PDHG for 3000 iterations

Figure 4 shows the images used to set up the optimization problem (36), in particular Fig. 4b which corresponds to *b* in said problem.

**Fig. 5:**
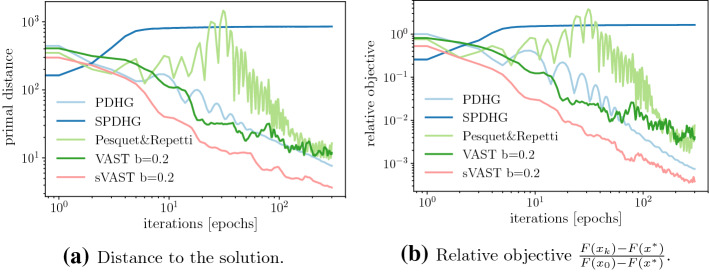
TV deblurring. Plots illustrating the performance of different methods

In Fig. 5 we see that while PDGH performs better in the deterministic setting, in particular in the later iteration, the stochastic variable smoothing method provides a significant improvement where sPDHG method seems not to converge. It is interesting to note that in this setting even the deterministic version of our algorithm exhibits a slightly chaotic behaviour. Although neither of the two methods is monotone in the primal objective function PDHG seems here much more stable.

### Matrix Factorization

In this section we want to solve a *nonconvex* and nonsmooth optimization problem of completely positive matrix factorization, see [16, 19, 27]. For an observed matrix $A \in {\mathbb {R}}^{d\times d}$ we want to find a completely positive low rank factorization, meaning we are looking for $x\in {\mathbb {R}}^{r\times d}_{\ge 0}$ with $r \ll d$ such that $x^{T}x=A$. This can be formulated as the following optimization problem

37 $$\begin{aligned} \min _{x\in {\mathbb {R}}^{r\times d}_{\ge 0}} \Vert x^{T}x - A \Vert _{1}, \end{aligned}$$

where $x^{T}$ denotes the transpose of the matrix *x*. The more natural approach might be to use a smooth formulation where $\Vert \cdot \Vert ^2_{2}$ is used instead of the 1-Norm we are suggesting. However, the former choice of distance measure, albeit smooth, comes with its own set of problems (mainly a non-Lipschitz gradient).

**Fig. 6:**
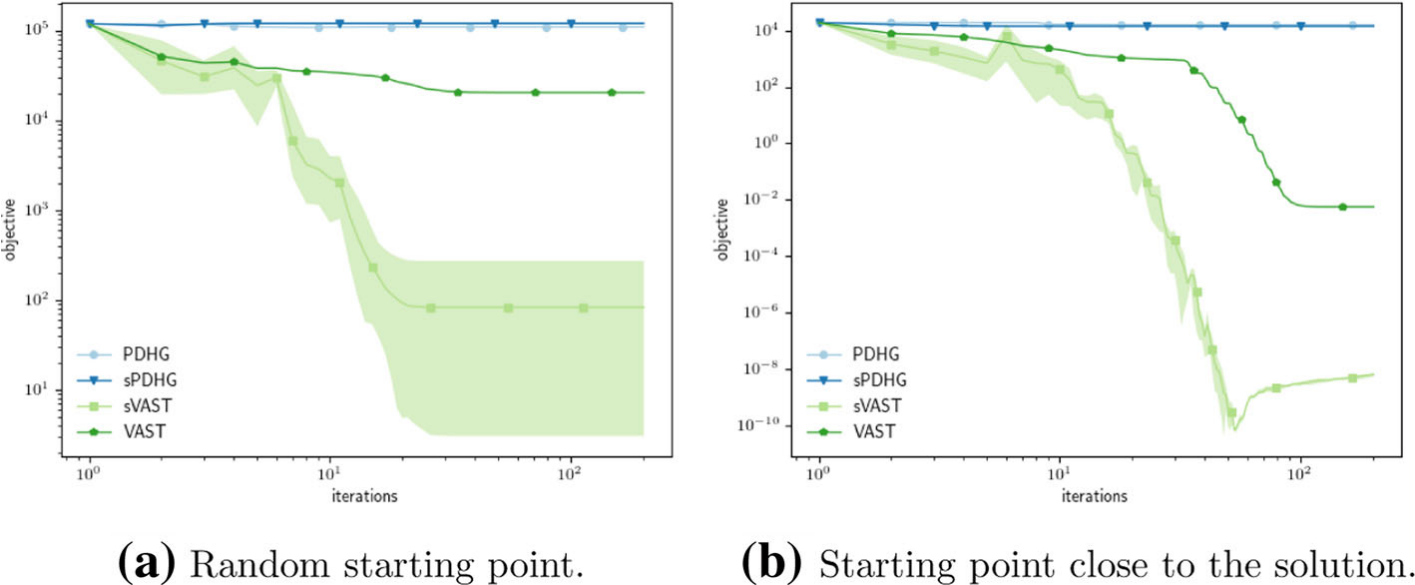
Comparison of the evolutions of the objective function values for different starting points. We run 40 epochs with 5 iterations each. For each epoch we choose the last iterate of the previous epoch as the linearization. For the stochastic methods we fix the number of rows (batch size) which are randomly chosen in each update a priori and count *d* divided by this number as one iteration. For the randomly chosen initial point we use a batch size of 3 (to allow for more exploration) and for the one close to the solution we use 5 in order to give a more accuracy. The parameter *b* in the variable smoothing method was chosen with minimal tuning to be 0.1 for both the deterministic and the stochastic version

The so called *Prox-Linear method* presented in [18] solves the above problem (37), by linearizing the smooth (${\mathbb {R}}^{d\times d}$-valued) function $x \mapsto x^{T}x$ inside the nonsmooth distance function. In particular for the problem

$$\begin{aligned} \min _{x} g(c(x)), \end{aligned}$$

for a smooth vector valued function *c* and a convex and Lipschitz function *g*, [18] proposes to iteratively solve the subproblem

38 $$\begin{aligned} x_{k+1} = \mathop {\mathrm {arg\, min}}\limits _{x}\left\{ g(c(x_{k}) + \nabla c (x_{k})(x-x_{k})) + \frac{1}{2t} \Vert x - x_{k} \Vert ^2_{2}\right\} \quad \forall k \ge 0, \end{aligned}$$

for a stepsize $t \le {(L_{g}L_{D\nabla c})}^{-1}$. For our particular problem described in (37) the subproblem looks as follows

39 $$\begin{aligned} x_{k+1} = \mathop {\mathrm {arg\, min}}\limits _{x \in {\mathbb {R}}^{r \times d}_{\ge 0}} \left\{ \Vert x_{k}^{T}x - A \Vert _{1} + \frac{1}{2}\Vert x - x_{k} \Vert ^2_{2}\right\} , \end{aligned}$$

and therefore fits our general setup described in (1) with the identification $f= \Vert \cdot - x_{k} \Vert ^2_{2} + \delta _{{\mathbb {R}}^{r\times d}_{\ge 0}}(x)$, $g=\Vert \cdot \Vert _{1}$ and $K = x_{k}^{T}$. Moreover, due to its separable structure, the subproblem (39) fits the special case described in (35) and can therefore be tackled by the stochastic version of our algorithm presented in Algorithm 4.3. In particular reformulating (38) for the stochastic finite sum setting we interpret the subproblem as

$$\begin{aligned} x_{k+1} = \mathop {\mathrm {arg\, min}}\limits _{x \in {\mathbb {R}}^{r \times d}_{\ge 0}} \left\{ \sum _{i=1}^{d}\left\Vert x_{k}^{T}[i,:]x - A[i,:] \right\Vert _{1} + \frac{1}{2}\Vert x - x_{k} \Vert ^2_{2}\right\} , \end{aligned}$$

where *A*[*i*,  : ] denotes the *i*-th row of the matrix *A* (Fig. 6).

In comparison to Sects. 5.1 and 5.2 a new aspect becomes important when evaluating methods for solving (38). Now, it is not only relevant how well subproblem (39) is solved, but also the trajectory taken in doing so as different paths might lead to different local minima. This can be seen in Fig. 6 where PDHG gets stuck early on in bad local minima. The variable smoothing method (especially the stochastic version) is able to move further from the starting point and find better local minima. Note that in general the methods have a difficulty in finding the global minimum $x_{true}\in {\mathbb {R}}^{3\times 60}$ (with optimal objective function value zero, as constructed $A: = x_{true}^{T}x_{true}\in {\mathbb {R}}^{60\times 60}$ in all examples).
